# Efficient Learning-Based Robotic Navigation Using Feature-Based RGB-D Pose Estimation and Topological Maps

**DOI:** 10.3390/e27060641

**Published:** 2025-06-15

**Authors:** Eder A. Rodríguez-Martínez, Jesús Elías Miranda-Vega, Farouk Achakir, Oleg Sergiyenko, Julio C. Rodríguez-Quiñonez, Daniel Hernández Balbuena, Wendy Flores-Fuentes

**Affiliations:** 1 Faculty of Engineering, Autonomous University of Baja California, Blvd. Benito Juárez, Mexicali 21280, Mexico; ederrodriguez@uabc.edu.mx (E.A.R.-M.); julio.rodriguez81@uabc.edu.mx (J.C.R.-Q.); dhernan@uabc.edu.mx (D.H.B.); 2 National Postdoctoral Fellowships, Ministry of Science, Humanities, Technology and Innovation, Insurgentes Sur, Mexico City 03940, Mexico; 3 Department of Electrical and Electronic Engineering, Tecnológico Nacional de México/IT Mexicali, Mexicali 21376, Mexico; elias.miranda@itmexicali.edu.mx; 4 Belive AI Lab, Belive.ai (former VusionGroup), 21 Rue Millevoye, 80000 Amiens, France; farouk.achakir@vusion.com; 5 Institute of Engineering, Autonomous University of Baja California, Calle Normal, Mexicali 21100, Mexico; srgnk@uabc.edu.mx

**Keywords:** robotic navigation, topological map, visual memory, RGB-D camera, point clouds, neural networks

## Abstract

Robust indoor robot navigation typically demands either costly sensors or extensive training data. We propose a cost-effective RGB-D navigation pipeline that couples feature-based relative pose estimation with a lightweight multi-layer-perceptron (MLP) policy. RGB-D keyframes extracted from human-driven traversals form nodes of a topological map; edges are added when visual similarity and geometric–kinematic constraints are jointly satisfied. During autonomy, LightGlue features and SVD give six-DoF relative pose to the active keyframe, and the MLP predicts one of four discrete actions. Low visual similarity or detected obstacles trigger graph editing and Dijkstra replanning in real time. Across eight tasks in four Habitat-Sim environments, the agent covered 190.44 m, replanning when required, and consistently stopped within 0.1 m of the goal while running on commodity hardware. An information-theoretic analysis over the Multi-Illumination dataset shows that LightGlue maximizes per-second information gain under lighting changes, motivating its selection. The modular design attains reliable navigation without metric SLAM or large-scale learning, and seamlessly accommodates future perception or policy upgrades.

## 1. Introduction

Autonomous navigation systems have become an essential area of research in robotics, enabling robots to move and operate in diverse environments without human intervention [1]. In many applications, such systems must navigate spaces where GPS signals are unavailable or unreliable, such as indoors areas [2,3]. Vision-based navigation systems, particularly those utilizing RGB-D sensors, have become a cornerstone technology in this area [4,5,6]. These systems enable robots to perceive their surroundings in 3D, offering a richer and more detailed understanding than traditional 2D imaging [7]. The depth data provided by RGB-D cameras enhances the robot’s ability to perform critical tasks such as obstacle avoidance, path planning, and complex scene understanding [8].

Traditional approaches to autonomous navigation, such as Simultaneous Localization and Mapping (SLAM), have made significant progress in addressing tasks like self localization and mapping [9]. Specifically, the integration of machine learning into these systems has introduced new possibilities for improving decision-making, such as path planning and obstacle avoidance, particularly in visual SLAM (vSLAM) systems [10]. Another approach is the use of machine learning models to interpret sensory data and make navigation decisions based on learned patterns, offering a data-driven alternative to classical rule-based systems [11,12,13,14]. These models can be combined with low-level controllers when a mobile robot navigates in presence of disturbance and uncertainties [15,16] or the system requires to adjust its feedback in real time [17].

Despite these advancements, several challenges persist in vision-based robotic navigation. One major issue is the computational complexity associated with processing high volumes of data from RGB-D sensors in real time [11,18,19]. Effective navigation requires not only recognizing and avoiding obstacles but also identifying optimal paths and making split-second decisions [20]. Traditional methods of point cloud registration used in SLAM techniques [21,22], such as the Iterative Closest Point (ICP) algorithm and its variants, often involve intensive computations to align and compare 3D models from different viewpoints [23]. These processes can be prohibitively slow for applications where timely response is critical.

The integration of visual memory, which allows robots to recall and recognize previously observed environments, further empowers navigation systems, enabling more adaptive and intelligent behavior in dynamic settings [24]. However, while visual memory systems enhance a robot’s navigation capabilities, they also introduce challenges in terms of memory management and data retrieval [25,26]. Efficiently indexing and accessing relevant spatial data from a robot’s memory without overloading the system’s computational resources is crucial for real-time performance [25,27]. Additionally, maintaining the accuracy and reliability of topological representation of the environment in constantly changing environments remains a significant hurdle, complicating the localization processes that are foundational for autonomous navigation [28].

### 1.1. Proposed System

**Figure 1:**
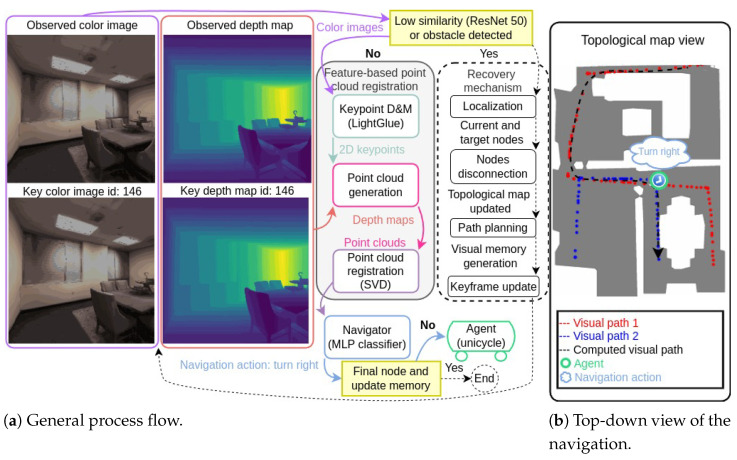
Overview of the proposed system during one iteration of autonomous navigation.

This work proposes a modular, vision-based navigation system that leverages RGB-D data for autonomous navigation, addressing the core tasks of localization, path planning, and obstacle avoidance. Central to the system is a machine learning-based navigator that interprets RGB-D data to suggest navigation actions, enabling real-time decision-making. The system organizes navigable paths using a topological map, where nodes represent keyframes (pairs of color images and depth maps) and edges correspond to navigable connections between them, facilitating efficient path planning. An overview of the proposed system during autonomous navigation is illustrated in Figure 1. The general process flow and the RGB-D data—including the observed color image and depth map (or “observed frame”) at the top, and the keyframe from the topological map (node 146) at the bottom—are included in Figure 1a. The similarity and obstacle detection modules assess whether the agent should initiate a recovery procedure. If recovery is unnecessary, the feature-based point cloud (or “PC” in the Figure) registration module estimates the relative pose and registration error through three steps: (1) keypoint detection and matching (or “D&M”) using LightGlue [29] (input: color images; output: 2D keypoint locations), (2) point cloud generation (input: depth maps and 2D keypoint locations; output: point clouds), and (3) point cloud registration using Singular Value Decomposition (SVD) to estimate the relative pose and registration error. Based on the relative pose estimation and registration error, the navigator—a Multi-Layer Perceptron (MLP) classifier—predicts the next navigation action. In the illustrated case, the predicted action is “turn right”. If the predicted action were “update memory” and the key RGB-D data corresponded to the final node, the navigation would conclude. The agent, modeled as a unicycle vehicle equipped with an RGB-D camera, executes the action and the process iterates. If the agent needs to recover, the system localizes the agent within the topological map using RGB-D data to determine the closest node by index (id). This node and the failed one are then disconnected from the topological map. Path planning is performed by finding a new path using Dijkstra’s algorithm over the updated topological map. This new path becomes the visual memory, consisting of a sequence of keyframes (pairs of color images and depth maps). The key RGB-D data is updated, and a new iteration of the process begins. Figure 1b presents a top-down view of the agent navigating the environment. Red and blue dots represent nodes of the topological map, containing two visual paths (1 and 2). The computed path (black arrow) is derived from the topological map and serves as a reference for the agent to reach the final key location using RGB-D data exclusively. The green circle marks the agent’s current position, with the predicted navigation action displayed above it.

The contributions of this paper are threefold:

1. A machine learning-based navigator that utilizes RGB-D data and feature-based point cloud registration for navigation decision-making.
2. The system employs a topological map to model the environment, integrating visual and geometric data from the RGB-D sensor to ensure reliable performance when searching for navigable paths.
3. The system was validated across four simulation environments using the Habitat-lab framework [30,31,32], demonstrating its ability to perform navigation tasks autonomously.

### 1.2. Paper Structure

The remainder of this paper is organized as follows. Section 2 reviews recent works in robotic navigation focusing on visual sensors. Section 3 and Section 4 detail the two main processes utilized by the system and present the system overview, respectively. In Section 5, the experiments conducted to select the best feature algorithm and assess the system across four different environments in the Habitat simulator are described. Then, the system’s main characteristics, advantages, limitations, and challenges are discussed in Section 6. Finally, this paper provides a brief conclusion in Section 7.

## 2. Related Work

This paper extends the problem of Visual Teach and Repeat (VTR) [33], which traditionally relies on path retracing based on visual memory, by incorporating a topological map that enables the system to perform localization, path planning, and obstacle avoidance. The rest of the section reviews related navigation methodologies, including SLAM, topological navigation, point cloud registration, reinforcement learning (RL), and vision-language navigation (VLN) systems. Then, the proposed system is introduced, and its capabilities—such as environment representation, localization, and navigation—are compared with those existing in the literature.

Conventional global information-based navigation methods, known as SLAM, are widely used in robotics and autonomous driving to build maps of the environment while simultaneously localizing the vehicle within them [34]. Recent advancements in SLAM techniques have incorporated RGB-D sensors, enabling more robust scene understanding and dense mapping. Some of these advancements focus on enhancing high-fidelity reconstructions and accelerating rendering processes by utilizing explicit volumetric representations and leveraging the structured regularities in environments [35,36,37]. Despite these improvements, SLAM often faces considerable computational challenges, including time-consuming map updates, reliance on accurate sensor data for local mapping, and difficulties in adapting to unforeseen changes in the environment [38,39,40].

In contrast to SLAM methods, topological map-based approaches [41,42] provide a more scalable solution, focusing on capturing the connectivity and layout of an environment rather than detailed metric information [27]. This method uses less computational resources and adapts more readily to changes within the environment [43,44]. For instance, Bista et al. [45] developed a topological navigation system for a resource-constrained robot that uses image memory for localization within a topological graph and has demonstrated reliable navigation in real-world environments without the need for accurate mapping. Similarly, Muravyev et al. [46] proposed NavTopo, a navigation pipeline that leverages topological maps for efficient path planning, achieving significant reductions in memory usage and computational demands compared to traditional metric-based methods. Additionally, Rodríguez et al. [47] proposed a navigation system, which generates navigable visual paths from aerial images of the scene, using photometric information and a topological map. Furthermore, Liu et al. [48] introduced a learning-based visual navigation framework that employs online topological maps enhanced with neural odometry and perceptual similarity measures, combining spatial proximity and perceptual resemblance. Their method also introduces a neural-based topological memory extraction technique (TMFT) for navigation decision-making, utilizing graph neural networks and a multi-factor attention mechanism to extract task-specific memory features, leading to improved navigation performance in complex environments. However, the approach has some limitations, as it depends on the quality of nodes representations (e.g., images), is susceptible to changes in the environment, and requires manual intervention for mapping [45].

One pivotal technique in global information-based navigation methods is the Iterative Closest Point (ICP) [49] algorithm which is particularly used for enhancing the accuracy of mapping in localization tasks and widely exploited in RGB-D-based systems [50]. This technique is often employed to align 3D point clouds, a crucial step in creating detailed and accurate maps from LIDAR and RGB-D sensors [22]. For instance, ref. [51] illustrates the implementation of a local ICP-SLAM aimed at improving computation time and localization accuracy by partitioning the environment into smaller segments. However, the ICP algorithm demands substantial computational resources as the environment size expands, potentially degrading localization and mapping performance [51]. Recent advances in optimization-based point cloud registration have introduced more efficient methods to address the high computational demands of ICP. For instance, ref. [52] proposed a novel algorithm that eliminates the need for complex matrix operations, significantly reducing computation time by up to 80%. Nevertheless, when the number of points becomes too large, using a feature-based algorithm can be less computationally expensive compared to the combinatory approach of optimization-based point cloud registration. For instance, Liu et al. [53] introduced a feature detection and matching technique that improves speed and accuracy by extracting feature points, applying Fast Point Feature Histogram (FPFH) features, and using the RANSAC algorithm to eliminate incorrect point pairs.

Reinforcement learning (RL) and end-to-end approaches in robotic navigation represent a significant shift from traditional methods, focusing on learning optimal actions directly through interactions with the environment. These methods utilize deep RL to train agents in simulations, enabling them to translate sensory inputs directly into control commands without the need for explicit environmental models [32]. By interacting with the environment and collecting sample data during navigation, mobile robots can learn effective strategies, leveraging deep learning’s perception abilities without requiring prior model information [19]. Particularly, RL has led to the emergence of various visual navigation techniques that integrate visual and depth cues from RGB-D data to build semantic augmented metric maps, combining environmental structure, appearance, and object semantics to enhance robotic navigation [54]. Additionally, some methods incorporate auxiliary tasks during training and utilize unsupervised topological simultaneous localization and mapping (SLAM) alongside memory networks to enable navigation in unseen environments without relying on pose information [27]. Despite these advancements, end-to-end learning-based methods face substantial challenges, including high sample complexity and low data efficiency, necessitating large amounts of training data [18,40]. Moreover, they often struggle with transferability and generalization, tending to memorize object locations and appearances specific to the training environments, which limits their applicability in real-world scenarios that differ from their training conditions [26,40].

In recent years, VLN systems have emerged as a significant development, enabling agents to navigate across 3D environments by following human instructions. These systems integrate visual and linguistic inputs, allowing for more natural human–robot interactions. However, current VLN agents often struggle with long-term planning and understanding complex scene geometries. To address these challenges, structured approaches like Structured Scene Memory (SSM), BEV Scene Graph (BSG), and Volumetric Environment Representation (VER) have been proposed, offering improved scene representation and planning capabilities by capturing comprehensive geometric and semantic information during navigation [12,13,55]. Specifically, VER quantizes the environment into structured 3D cells, enabling better grounding of instructions in the 3D context and enhancing navigation performance in complex environments [55]. Additionally, multitask agents like VIENNA are designed to handle multiple embodied navigation tasks, including VLN, using a unified model for decision-making across different input modalities [14]. Furthermore, LANA introduces a language-capable navigation agent that not only follows navigation instructions but also generates route descriptions to humans by simultaneously learning instruction following and generation tasks with a single model, enhancing explainability and human–robot communication in VLN [56]. Despite these advancements, VLN still struggles with effectively integrating multi-modal information, without escalating system complexity, and with mitigating hallucination issues where agents may generate non-existent objects or inaccurate instructions [57,58].

The proposed vision-based navigation system integrates a machine learning-based navigator with a topological map and feature-based point cloud registration, enabling autonomous navigation using RGB-D data only. Unlike traditional SLAM methods, which often face computational challenges and difficulties adapting to environmental changes [38,39,40], the system leverages a topological map that captures the environment’s connectivity without requiring detailed metric information, significantly reducing computational overhead. Furthermore, the system incorporates both visual and geometric criteria when establishing connections between nodes. Specifically, it uses cosine similarity of ResNet50-embedded vectors for the visual criterion and considers the agent’s kinematic constraints for the geometric criterion. This dual-criteria method improves the reliability of path planning and execution compared to methods that rely solely on visual information [59]. Moreover, the system distinguishes itself from point cloud registration techniques that rely on computationally intensive algorithms like ICP by utilizing feature-based point cloud registration with LightGlue and SVD. This approach reduces computational demands, making it suitable for real-time applications on resource-constrained platforms. Additionally, unlike end-to-end reinforcement learning approaches that require extensive training data and struggle with generalization to unseen environments [18,40], the proposed modular system achieves efficient navigation without the high sample complexity, offering a practical and adaptable solution for autonomous navigation tasks.

## 3. Visual and Geometric Processes

This section delves into two key concepts of the proposed navigation system: image similarity and feature-based point cloud registration. The similarity process ensures that the content of two color images is sufficiently alike before further processing. This is achieved by comparing embedded vectors, extracted using ResNet50 [60], through cosine similarity. This process, along with others, contributes to generating visual memories during model training, connecting nodes in the topological map during the second phase, and localizing the agent and activating the recovery module during autonomous navigation. The feature-based point cloud registration estimates the relative pose between two point clouds, enabling the agent to determine its position and orientation relative to a key location using only RGB-D data.

### 3.1. Image Similarity

The image similarity process implemented in the proposed system is based on the comparison of embedded feature vectors derived from two color images. The main goal is to quantify how visually similar the images are before further processing, which is essential for tasks such as localization, navigation, and recovery and nodes connection in the topological map.

For this purpose, ResNet50 is utilized [60], a well-established deep convolutional neural network that has been pre-trained on the ImageNet dataset [61]. The ResNet50 model is specifically designed to extract robust, high-level features from images. One of the motivations behind selecting ResNet50 is its depth and architecture, which allow for the capture of intricate visual patterns while addressing the vanishing gradient problem through residual connections. These properties make it suitable for tasks involving the extraction of discriminative features from color images.

#### 3.1.1. Feature Embeddings

In the implemented process, the final fully connected (FC) layer of ResNet50 is removed, leaving the network as a feature extractor that outputs a 2048-dimensional embedded vector for each input image. Given two input images, the observed color image $I_{o}$ and the key color image $I_{\mathrm{key}}$, the goal is to compute their similarity by first extracting the feature embeddings, $e_{o}$ and $e_{\mathrm{key}}$, using the truncated ResNet50 model. The feature extraction process can be represented as follows:

(1) $$e_{o}=\mathrm{ResNet}50\left(I_{o}\right),\;e_{\mathrm{key}}=\mathrm{ResNet}50\left(I_{\mathrm{key}}\right)$$

#### 3.1.2. Cosine Similarity

Once the feature embeddings are obtained, the similarity *s* score between the two color images is computed using cosine similarity. Cosine similarity is widely used for comparing feature embeddings in tasks involving image similarity, recommendation systems, and other query-based applications, as it measures the orientation between vectors in the embedding space, disregarding their magnitude [62]. This makes it especially effective when working with normalized feature embeddings, such as those produced by ResNet50 after standard pre-processing and normalization [63]. It measures the cosine of the angle between two vectors in a high-dimensional space, yielding a similarity score in the range of [−1,1], where 1 indicates perfect similarity, and −1 indicates complete dissimilarity. The cosine similarity between two feature embeddings $e_{o}$ and $e_{\mathrm{key}}$ is calculated as follows:

(2) $$s\left(I_{o},I_{\mathrm{key}}\right)=\frac{e_{o}\cdot e_{\mathrm{key}}}{∥e_{o}∥∥e_{\mathrm{key}}∥}$$

Here, $e_{o}\cdot e_{\mathrm{key}}$ represents the dot product of the two feature embeddings, and $∥e_{o}∥$ and $∥e_{\mathrm{key}}∥$ are their respective Euclidean norms. This equation results in a scalar value or score $s_{o,\mathrm{key}}=s\left(I_{o},I_{\mathrm{key}}\right)$ that reflects the similarity between the two color images based on their extracted embeddings.

In summary, the combination of ResNet50 as a feature extractor and cosine similarity as a comparison metric provides an effective mechanism for quantifying image similarity using color images. However, the image similarity process alone does not guarantee path navigability, as the latter often involves additional constraints, such as those imposed by the vehicle’s kinematics. In such cases, it becomes necessary to estimate the relative pose between the scenes represented by these color images once the content of two color images has been confirmed to be similar. This requires processing the depth information, contained in the depth maps, associated with the color images to generate point clouds. The feature-based point cloud registration process is then applied to align these point clouds, allowing the system to estimate the agent’s relative position and orientation with respect to key locations in the environment. This geometric understanding complements the image similarity step, enabling the agent to navigate accurately even in three-dimensional spaces.

### 3.2. Feature-Based Point Cloud Registration

Point cloud, generated from LiDAR and RGB-D sensors, is the primary data format to represent the 3D world [23]. The proposed system utilizes this 3D data, specifically color images and depth maps from its RGB-D sensor, to determine the relative position and orientation between a pair of key and observed color images and depth maps, called keyframes and observed frames, respectively, in tasks such as localization and navigation. A common approach, known as optimization-based registration, involves generating point clouds directly from color images and depth maps, followed by estimating their relative pose using the ICP algorithm, its variants [23], or more recent optimized methods like FA3R [52]. However, this approach is computationally intensive, as it relies on a combinatorial search to identify corresponding points between two point clouds. In contrast to this approach, the proposed system employs a feature-based method to estimate the relative pose between two point clouds. This method consists in three steps: keypoint detection and matching, point cloud generation, and point cloud registration. Generally speaking, the process begins by detecting distinctive keypoints in the color images associated with the depth maps, and then matching these keypoints across images using keypoint detection and matching methods. With these matched points, and their corresponding depth values extracted from their corresponding depth maps, the process generates two reduced point clouds, and the relative pose is then estimated by applying SVD [64], avoiding an iterative optimization process. This process is illustrated at the center of Figure 1 when executed during one iteration of the autonomous navigation phase.

#### 3.2.1. Keypoint Detection and Matching

Given two color images, $I_{o}$ and $I_{\mathrm{key}}$, the process begins by detecting and matching keypoints $u_{o}=\left(u_{o},v_{o}\right)$ and $u_{\mathrm{key}}=\left(u_{\mathrm{key}},v_{\mathrm{key}}\right)$ in each color image using a point feature algorithm. A detailed description of this algorithm is provided in [65].

#### 3.2.2. Point Cloud Generation

The projection of a keypoint p∈P and p=(x,y), where *x* and *y* are the normalized image coordinates, from its pixel coordinates (u,v) to its corresponding 3D coordinates P=(X,Y,Z) in the camera’s reference frame using the pinhole camera model is described as follows. Given the intrinsic camera parameters matrix:

(3) $$K=\left[\begin{array}{ccc} f_{x} & 0 & c_{x}\\ 0 & f_{y} & c_{y}\\ 0 & 0 & 1 \end{array}\right]$$

where $f_{x}$ and $f_{y}$ are the focal lengths along the x and y axes of the image plane, respectively, and $\left(c_{x},c_{y}\right)$ is the principal point, or the coordinates of the image center.

The image coordinates (u,v) are first transformed to normalized image coordinates (x,y) using the inverse of the intrinsic parameters matrix:

(4) $$\left[\begin{array}{c} x\\ y\\ 1 \end{array}\right]=K^{-1}\cdot \left[\begin{array}{c} u\\ v\\ 1 \end{array}\right].$$

Hence, *x* and *y* here represent the normalized coordinates in the image plane:

(5) $$x=\frac{u-c_{x}}{f_{x}},\;y=\frac{v-c_{y}}{f_{y}}.$$

For a projected keypoint P=(X,Y,Z), given the depth *Z* from the depth map *D*, the *X* and *Y* coordinates are calculated by scaling the normalized image coordinates *x* and *y* by *Z*:

(6) X=Z·x,Y=Z·y

This maintains the spatial consistency between the 2D image and its corresponding 3D representation, where *Z* in the 3D coordinate system represents the depth of the point (u,v) from the camera along the optical axis.

This process is applied to both frames $\left(I_{o},D_{o}\right)$ and $\left(I_{\mathrm{key}},D_{\mathrm{key}}\right)$ to generate the observed and key point clouds $P_{o}$ and $P_{\mathrm{key}}$, respectively, where P∈P.

#### 3.2.3. Point Cloud Registration

The relative pose ^key^$\xi_{o}\in \mathrm{SO}\left(3\right)$ in 6 Degrees of Freedom (DoF) between two camera frames O and K observing a point cloud P, can be expressed by:

(7) $${\hat{P}}_{\mathrm{key}}{=\;}^{\mathrm{key}}M_{o}{\hat{P}}_{o},$$

where ${\hat{P}}_{\mathrm{key}}$ and ${\hat{P}}_{o}$ are the homogeneous coordinates of the points $P_{\mathrm{key}}$ and $P_{o}$, respectively, in the point clouds observed from camera frames K and O. The matrix ^key^$M_{o}$ represents the transformation from frame O to frame K and is composed of a rotation matrix R∈SO(3) and a translation vector $t\in R^{3}$. The matrix ^key^$M_{o}$ can be expressed as:

(8) .keyMo=Rt01.

To estimate the rotation *R* and *t* from a set of corresponding points in P observed in O and K, SVD can be applied. First, the centroids of the corresponding points in both frames are computed:

(9) $${\bar{P}}_{o}=\frac{1}{n}\sum_{i=1}^{n}P_{o_{i}},\;{\bar{P}}_{\mathrm{key}}=\frac{1}{n}\sum_{i=1}^{n}P_{{\mathrm{key}}_{i}},$$

where *n* is the number of corresponding points. The cross-covariance matrix between the point sets is then formed:

(10) $$H=\sum_{i=1}^{n}\left(P_{o_{i}}-{\bar{P}}_{o}\right){\left(P_{{\mathrm{key}}_{i}}-{\bar{P}}_{\mathrm{key}}\right)}^{⊤}.$$

The SVD of *H* is computed to yield:

(11) $$U\Sigma V^{⊤}=\mathrm{SVD}\left(H\right).$$

The rotation matrix *R* is determined by:

(12) $$R=VU^{⊤},$$

and the translation vector *t* is computed as:

(13) $$t={\bar{P}}_{\mathrm{key}}-R{\bar{P}}_{o}.$$

After estimating the transformation parameters *R* and t, the next step is to compute the Root Mean Square Error (RMSE) between the transformed point cloud and the key point cloud $P_{\mathrm{key}}$. This metric error is calculated by applying the transformation to each point in the observed point cloud $P_{o}$ and then computing the Euclidean distance to the corresponding point in $P_{\mathrm{key}}$. The RMSE is then given by:

(14) $$e=\sqrt{\frac{1}{n}\sum_{i=1}^{n}{∥\left(RP_{o_{i}}+t\right)-P_{{\mathrm{key}}_{i}}∥}^{2}},$$

where *n* is the number of points, $P_{o_{i}}$ are the points in the observed point cloud, and $P_{{\mathrm{key}}_{i}}$ are the points in the key point cloud. This error provides a quantitative measure of the accuracy of the estimated transformation by reflecting the average distance errors between the transformed points in $P_{o}$ and those in $P_{\mathrm{key}}$.

Lastly, the rotation matrix (Equation (12)) is used in its quaternion representation q, following the Hamiltonian convention [66], by the navigation policy to represent the rotation.

The complete process is detailed in Appendix A, Algorithm A1.

## 4. System Overview

The system operates in three main phases: model training, topological map generation, and autonomous navigation. During the training phase, the agent is manually controlled to collect pairs of color images and depth maps (or observed frames), through four different paths in a given environment. For each path, a visual memory is built by sequentially comparing the cosine similarity of the ResNet50-embedded vectors [60] of the previously collected color images. The agent then is manually controlled to retrace each path using the visual memory as a reference, collecting observed frames and keyframes (those stored in the visual memory) at each iteration, along with navigation actions. These RGB-D data and commands are used to train several MLP models with varying architectures and hyperparameters. The input to the MLP consists of the results from feature-based point cloud registration, including relative translation, rotation (in quaternion), and registration error. The ideal feature matching algorithm should exhibit a good balance between the number of correct matches and time efficiency. The MLP with the highest F-1 score is selected and implemented as the navigator for deployment. In the topological map generation phase, the agent collects new RGB-D data and selects a group of them based on a visual criterion, using cosine similarity to ensure image content similarity. Additionally, a geometric pose criterion based on the agent’s unicycle kinematics prevents the connection of nodes if movement would violate constraints, such as moving backward or sideways. When both conditions are met, the nodes are connected to form the topological map. In the autonomous navigation phase, the agent uses the navigator and the generated topological map to localize itself, compute an optimal path using Dijkstra’s algorithm [67], and follow this path based on real-time observations. The system continuously evaluates the similarity between the the observed frames and the keyframes while estimating the agent’s relative pose. If the agent encounters obstacles, it updates the map and recalculates the path to ensure successful navigation.

The rest of the section will explain step by step each phase as well as their modules.

### 4.1. Model Training

**Figure 2:**
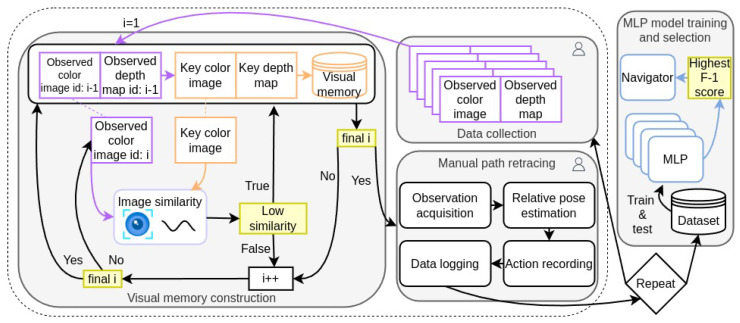
Overview of the model training phase which includes: data collection, visual memory construction, manual path retracing, and MLP model training and selection.

The model training phase is dedicated to developing a high-level decision-making neural network that can suggest appropriate navigation actions based on the agent’s observations and its relative position within the environment. This phase is illustrated in Figure 2  and begins with the data collection process (top center), where RGB-D data is collected by manually controlling the agent through the environment. Next, during the visual memory construction process (left), a visual memory is generated by sequentially measuring the image similarity between each observed color image $I_{i}$ and the current key color image. If the similarity score is low, the previous frame is appended to the visual memory; the first and last frames are always included directly. Then, the manual path retracing process (bottom center) involves manually guiding the agent to retrace the path using the visual memory as a reference. This process consists of iteratively acquiring observed RGB-D data, computing the relative pose with respect to the keyframe, applying a navigation action, and logging both the output of the relative pose estimation and the navigation action until the navigation is completed (i.e., when the navigation action is finish). These steps are repeated few times (e.g., four times) to gather sufficient data. Finally, in the MLP model training and selection process (right), the dataset collected from the previous steps is used to train and evaluate multiple MLP models with different architectures and parameters. These models act as classifiers to predict the navigation action based on the output of the relative pose estimation. The model that achieves the highest F-1 score is selected and implemented as the navigator during the autonomous navigation phase.

#### 4.1.1. Data Collection

The goal of this step is to manually guide the agent through the environment on the most reasonable trajectories according to human logic, acquiring the RGB-D data with predefined sampling period. Then, the data will serve to construct visual memories which will serve as reference in a later step within the model training phase. When traversing a given path, the robot observes a color image $I_{o_{i}}$ and a corresponding depth map $D_{o_{i}}$, where *i* is the iteration, forming a sequence of observations ${\left\{\left(I_{o_{i}},D_{o_{i}}\right)\right\}}_{i=1}^{N}$, where *N* is the total number of iteration steps.

#### 4.1.2. Visual Memory Construction

For each traversed path, a visual memory V is constructed to represent key locations within the environment using RGB-D data only. The process initiates by extracting a high-dimensional embedding of the first frame using a pre-trained ResNet50 network (Equation (1)), designating it as the initial keyframe and adding it to the visual memory V. As subsequent frames are processed, each frame’s embedding is computed and compared to the current keyframe’s embedding using a similarity measure, such as cosine similarity. If the similarity score *s*, computed from Equation (2), falls below a predefined threshold $\epsilon_{\mathrm{select}}$, indicating a significant deviation from the keyframe, the process updates the keyframe to the preceding frame and appends this new keyframe to V. This selective inclusion ensures that the visual memory retains only the most representative and diverse frames, thereby reducing redundancy and optimizing memory usage. Additionally, to guarantee comprehensive coverage of the entire sequence, the process explicitly adds the final frame to V if it has not been included through the iterative process. The complete algorithm for visual memory construction is depicted in Appendix A, Algorithm A2.

#### 4.1.3. Manual Path Retracing

After constructing the visual memory V for each path (as described in Section 4.1.2), the agent is manually guided to retrace these paths using V as a reference. The primary objective of this step is to collect a dataset that maps the agent’s observations and estimated relative poses to the navigation actions required to progress along the path. This dataset will be instrumental in training the MLP classifier detailed later.

During the retracing process, the following operations are carried out at each iteration *i*:

1. Observation Acquisition: Capture the observed frame $\left(I_{o_{i}},D_{o_{i}}\right)$ at the current location.
2. Relative Pose Estimation: Estimate the relative translation $t_{i}$, rotation $q_{i}$ (expressed as a quaternion) and registration error $e_{i}$ between the observed frame $\left(I_{o},D_{o}\right)$ and the current keyframe $\left(I_{\mathrm{key}},D_{\mathrm{key}}\right)$ in V as depicted in Section 3.2. For the sake of simplicity, the system considers that the agent’s frame A and camera frame C coincide.
3. Action Recording: Apply and record the navigation action $a_{i}$ executed to move towards the next keyframe. Actions are discrete commands such as:
  - *Move forward* for a fixed length. This action is applied when the user considers that the location depicted by the keyframe $\left(I_{\mathrm{key}},D_{\mathrm{key}}\right)$ is in front of the agent.
  - *Turn left* or *turn right* by a fixed angle. These actions are applied in two situations: (1) when the user considers that the agent should rotate left or right to align itself with the location depicted in keyframe $\left(I_{\mathrm{key}},D_{\mathrm{key}}\right)$ or (2) when the agent requires to rotate before moving forward because lateral movement is not possible.
  - *Update memory* by defining the next keyframe in V as the current one. This action is applied when the user considers that the location depicted in the keyframe $\left(I_{\mathrm{key}},D_{\mathrm{key}}\right)$ has been reached and the keyframe is not the last one in the visual memory V. If that is the case, the *finish* action should be applied instead to conclude the operation.
  Recording the navigation actions during retracing is crucial for supervised learning, as they capture the expert decisions required to navigate from the current position to the next key position depicted by the keyframe. These actions serve as labels in the training dataset, enabling the model to learn and replicate the expert’s navigation strategy. It is important to note that, while the action *finish* signifies the end of the manual operation, any frames associated with this action are excluded from the training data to prevent biasing the model with terminal states.
4. Data Logging: Store the collected data $\left\{t_{i},q_{i},e_{i},a_{i}\right\}$ for training purposes.

The final dataset D compiled from the retracing process consists of input–label pairs:

(15) $$D={\left\{\left(X_{i},a_{i}\right)\right\}}_{i=1}^{M},$$

where $X_{i}=\left(t_{i},q_{i},e_{i}\right)$ represents the input data and *M* is the total number of iterations during manual retracing. This dataset, which aims to replicate the decision-making process observed during manual navigation, is subsequently split into training and testing subsets for model development and evaluation.

#### 4.1.4. MLP Model Training and Selection

The collected dataset D from the manual path retracing phase serves as the foundation for training an MLP classifier that will act as the agent’s navigator. The objective is to train a model that can predict the appropriate navigation action based on the estimated relative pose and registration error.

The navigation decision-making problem is formulated as a multi-class classification task. Given an input vector $X_{i}=\left(t_{i},q_{i},e_{i}\right)$, the goal is to predict the corresponding action $a_{i}$ from a set of discrete actions A:

(16) $$f_{\theta }:X_{i}\mapsto a_{i},\;a_{i}\in A,$$

where $f_{\theta }$ represents the MLP classifier parameterized by θ. A group of MLP models with varying architectures were trained and evaluated to explore the impact of different hyperparameters on performance. The variations include evaluating different activation functions, and exploring different optimizers.

After training, models were evaluated on the test set. Precision, which measures the proportion of correctly identified positive instances among all predicted positives, is defined as:

(17) $$\mathrm{Precision}=\frac{\mathrm{TP}}{\mathrm{TP}+\mathrm{FP}},$$

where TP and FP represent true positives and false positives, respectively. Recall, which measures the proportion of correctly identified positive instances among all actual positives, is defined as:

(18) $$\mathrm{Recall}=\frac{\mathrm{TP}}{\mathrm{TP}+\mathrm{FN}},$$

where FN represents false negatives. The F-1 score, which balances precision (Equation (17)) and recall (Equation (18)), is defined as:

(19) $$F1-\mathrm{score}=2\times \frac{\mathrm{Precision}\times \mathrm{Recall}}{\mathrm{Precision}+\mathrm{Recall}}.$$

The model that achieved the highest F-1 score was selected for deployment as the navigator.

### 4.2. Topological Map Generation

The second phase involves manually controlling the agent along new paths while recording RGB-D data at each iteration similar to Section 4.1.1. From each traversed path, a visual memory V is constructed by selecting a group of keyframes $\left\{\left(I_{\mathrm{key}},D_{\mathrm{key}}\right)\right\}$ as detailed in Section 4.1.2. Then, the topological map is defined as a directed graph G=(N,E), where *N* represents the set of nodes and *E* the set of edges. Each node $N_{i}\in N$ corresponds to a keyframe $\left(I_{{\mathrm{key}}_{i}},D_{{\mathrm{key}}_{i}}\right)$. An edge $E_{i,j}$ is established from node $N_{i}$ to node $N_{j}$ if both visual and geometric criteria are satisfied.

#### 4.2.1. Visual Criterion

The visual criterion ensures that the content between two color images $I_{o}$ and $I_{\mathrm{key}}$ is similar. Given two different keyframes $\left(I_{{\mathrm{key}}_{i}},D_{{\mathrm{key}}_{i}}\right)$ and $\left(I_{{\mathrm{key}}_{j}},D_{{\mathrm{key}}_{j}}\right)$, the visual criterion is satisfied if the similarity score is greater than the visual threshold:

(20) $$s_{i,j}>\epsilon_{\mathrm{visual}},$$

where the similarity score $s_{i,j}$ is computed from $I_{{\mathrm{key}}_{i}}$ and $I_{{\mathrm{key}}_{j}}$ as depicted in Section 3.1.

#### 4.2.2. Geometric Criteria

The geometric criteria ensure that the agent can move from keyframe $\left(I_{i},D_{i}\right)$ to keyframe $\left(I_{j},D_{j}\right)$ given its kinematic constraints. Since the agent cannot move backward, the forward displacement along the *z*-axis from the translation vector t (as defined in Equation (13)) must exceed a threshold $\epsilon_{z}$:

(21) $$t_{z}>\epsilon_{z},$$

this condition is called the backward constraint.

Additionally, due to the agent’s inability to move laterally without turning, a lateral constraint is imposed. The lateral displacement is defined as:

(22) $$d_{\mathrm{lateral}}=\sqrt{t_{x}^{2}+t_{y}^{2}},$$

where $t_{x}$ and $t_{y}$ are the components of the translation vector t. The ratio of lateral to forward displacement must be less than a lateral threshold $\epsilon_{\mathrm{lateral}}$:

(23) $$\frac{d_{\mathrm{lateral}}}{t_{z}}<\epsilon_{\mathrm{lateral}}.$$

If either the lateral displacement constraint or the backward constraint is not satisfied, nodes $N_{i}$ and $N_{j}$ are not connected.

**Figure 3:**
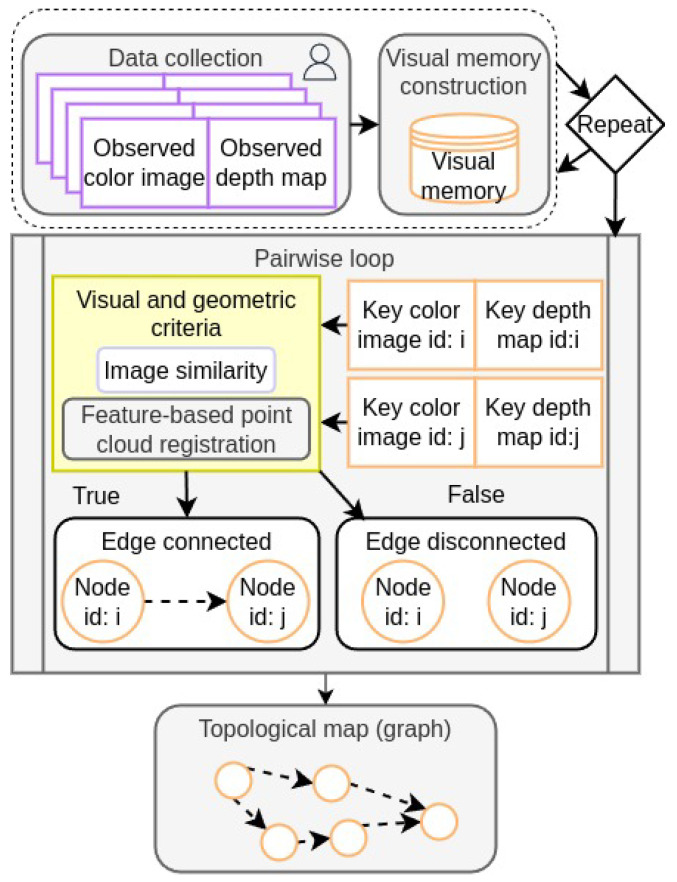
Overview of the topological map generation.

The resulting graph *G*, with nodes and edges defined by the aforementioned criteria, constitutes the topological map used during the autonomous navigation phase. This map enables the computation of feasible paths between nodes using Dijkstra’s algorithm, facilitating efficient and reliable navigation within the environment. The generation of the topological map is detailed in Appendix A, Algorithm A3. Figure 3 illustrates this phase that begins by manually controlling the agent through new paths and constructing one visual memory for each path. Then, a node is associated to each keyframe in the visual memories. Later, the visual and geometric criteria are evaluated for every combination of two different nodes associated to their respective keyframes in a pairwise loop. If the criteria is met, the connection between these two nodes is established (the edge is created); otherwise, the nodes are disconnected. Finally, the topological map is defined as a directed graph whose nodes are associated with keyframes and their edges as navigable paths.

### 4.3. Autonomous Navigation

The autonomous navigation phase leverages the trained MLP navigator and the topological map generated in previous phases to enable the agent to navigate from its current location to a specified goal without human intervention. This phase encompasses localization, path planning, real-time decision-making, obstacle detection, and recovery mechanisms to ensure robust navigation within the environment. Figure 1 exemplifies one iteration of this phase after initial positioning.

#### 4.3.1. Localization and Initial Positioning

At the beginning of the autonomous navigation, or whenever the recovery module is activated, the agent localizes itself within the topological map by identifying the closest and frontally located keyframe. Localization is achieved by estimating the relative pose between the agent’s current RGB-D observation $\left(I_{o},D_{o}\right)$ and each keyframe $\left(I_{\mathrm{key}},D_{\mathrm{key}}\right)$ in the topological map using the feature-based point cloud registration method detailed in Section 3.2.

A keyframe is considered frontal if the forward component of the translation vector *t* satisfies the following:

(24) $$t_{z}>\epsilon_{z},$$

where $t_{z}$ is the *z* component of t, indicating that the keyframe is in front of the agent.

To determine the closest keyframe, a cost function *C* is computed for each frontal keyframe:

(25) $$C=\left\lceil \frac{\left|t\right|}{d_{\mathrm{step}}}\right\rceil +\left\lceil \frac{\left|\phi \right|}{\theta_{\mathrm{step}}}\right\rceil ,$$

where:

- |t| is the magnitude of the translation vector obtained from the relative pose estimation (see Equation (13)).
- ϕ is the yaw angle extracted from the rotation matrix *R* (see Equation (12)).
- $d_{\mathrm{step}}$ is the predefined translation step size (e.g., 0.1,m).
- $\theta_{\mathrm{step}}$ is the predefined rotation step size (e.g., $1^{\circ }$).

The keyframe with the minimal cost *C* is selected as the agent’s initial position in the topological map. This approach ensures that the agent begins navigation from the most feasible and closest known location. In case of tie, the keyframe with the lowest index is selected. The localization module is detailed in Appendix A, Algorithm A4.

#### 4.3.2. Path Planning Using Dijkstra’s Algorithm

With the initial and goal nodes defined, the agent computes the optimal path using Dijkstra’s algorithm applied to the topological map G=(N,E) constructed in Section 4.2. The graph is a directed graph without weights, indicating navigable paths between keyframes.

Since global metric information is unknown and edge weights are uniform, the shortest path is the one with the least number of nodes. The agent uses Dijkstra’s algorithm to find this path, ensuring efficient navigation from the current location to the goal node.

#### 4.3.3. Recovery Mechanism

The agent employs a recovery mode to handle unexpected situations that prevent it from following the planned path. Two primary conditions trigger the recovery mechanism: obstacle detection and low visual similarity. In both cases, the agent updates the topological map by disconnecting edges and replans the path to the goal using Dijkstra’s algorithm from its current location. Specifically, the recovery mechanism consists on these steps:

1. Topological Map Update: Disconnect the edge between the current node and the next node in the path within the topological map *G*, effectively marking the path as obstructed by a static obstacle.
2. Path Replanning: Recompute a new path to the goal node using Dijkstra’s algorithm on the updated topological map.
3. Visual Memory Generation: Generate a new visual memory V as the sequence of keyframes associated to the previously computed path.

##### Obstacle Detection

The agent continuously monitors for obstacles not represented in the topological map using the depth data $D_{o}$ from its current observation. An obstacle is detected in the frontal region when any point within a predefined threshold distance $\epsilon_{\mathrm{distance}}$ is found directly ahead. If a frontal obstacle is detected while the suggested action $a_{i}$ is move forward, the agent enters recovery mechanism. This process allows the agent to dynamically adapt to changes in the environment and avoid obstacles without reversing or retracing its path, in accordance with its unicycle kinematic constraints.

##### Low Visual Similarity

At each iteration, the agent evaluates the visual similarity $s_{i}$ between its current observation $\left(I_{o},D_{o}\right)$ and the current keyframe $\left(I_{\mathrm{key}},D_{\mathrm{key}}\right)$ using the cosine similarity function defined in Equation (2) (Section 3.1). If the similarity falls below the visual threshold $\epsilon_{\mathrm{nav}}$, the agent detects a significant discrepancy between expected and observed visual data, i.e., low similarity, and enters recovery mechanism.

#### 4.3.4. Navigation Decision-Making

During navigation, the agent follows the computed path by sequentially moving towards the keyframes along the path. At each iteration *i*, the agent performs the following steps:

1. Observation Acquisition: Capture the current RGB-D observation $\left(I_{o},D_{o}\right)$.
2. Relative Pose Estimation: Estimate the relative translation $t_{i}$, rotation $q_{i}$, and registration error $e_{i}$ between $\left(I_{o},D_{o}\right)$ and the current keyframe $\left(I_{\mathrm{key}},D_{\mathrm{key}}\right)$ using the feature-based point cloud registration method (Section 3.2).
3. Action Prediction: Input the relative pose $\left(t_{i},q_{i},e_{i}\right)$ into the trained MLP navigator (Equation (16)) to predict the suggested navigation action $a_{i}$.
4. Action Execution: Execute the suggested action $a_{i}$, unless the agent’s recovery mechanism is activated due to obstacle detection *or* low visual similarity.
5. Memory Update: If the action $a_{i}$ is “*update memory*” and the current keyframe is not the last one in the path, the agent updates the keyframe to the next one in the path. If it is the last keyframe, the agent concludes the navigation.

The autonomous navigation phase is summarized in Appendix A, Algorithm A5, and Figure 1 exemplifies the process during one iteration.

## 5. Experimental Setup and Evaluation

The goal of the experimentation is two-fold: (1) to identify and select the best feature algorithm based on specific criteria, such as illumination robustness, low inference time, and keypoint matching accuracy for implement it to the system and (2) to evaluate the proposed system. To this end, two evaluations were considered: feature algorithm assessing and navigation tasks.

The experimentation was conducted on an OMEN HP Gaming Laptop equipped with a 13th Gen Intel processor with 13GB of RAM and an NVIDIA GeForce RTX 4050 GPU, running Ubuntu 18.04 and Python 3.9 for compatibility reasons. For the first evaluation, the Multi-Illumination Images in the Wild dataset [68] was employed, while four virtual environments from Habitat [31,32] were utilized for the second evaluation. Specifically, the Multi-Illumination Dataset was chosen because it contains scenes whose content is constant, but the illumination varies. The data in the Multi-Illumination Dataset was collected using a setup comprising a Sony α6500 mirrorless camera equipped with a 24 mm lens, offering a $52^{\circ }$ horizontal and $36^{\circ }$ vertical field of view. Illumination was provided by a Sony HVL-F60M external flash unit, precisely controlled via two servo motors to alter the flash direction across 25 predetermined positions uniformly distributed over the upper hemisphere [68]. Four traditional feature algorithms—AKAZE, BRISK, ORB [69], and SIFT [70]—and two learned-feature algorithms—SuperGlue [71] and LightGlue [29]—were evaluated in the first experiment and the best one was implemented to evaluate the system performance in the second experiment. The rest of the section details the setup and the experimental evaluation.

### 5.1. Information-Theoretic Evaluation of Feature Algorithms

Feature matching can be interpreted as a noisy channel that “transmits’’ geometric information from the image domain to the descriptor domain. Using Shannon’s seminal framework [72] and the treatment of entropy and mutual information in [73], six candidate algorithms —AKAZE, BRISK, ORB, SIFT, SuperGlue, and LightGlue— are assessed from an *information-theory* (IT) point of view instead of purely geometric error.

Given the 25×25 illumination grid of the Multi-Illumination Dataset, we treat every image pair as one use of a binary channel whose output Z∈{0,1} indicates a successful correspondence. A match is counted as true positive when the distance between the paired keypoints is below the 10-pixel; otherwise, it is a false positive. Three IT quantities are computed:

1. **Confidence entropy:** $H_{\mathrm{conf}}=-p\log_{2}p-\left(1-p\right)\log_{2}\left(1-p\right)$ averaged over all 625 pairs, where $p=\frac{\mathrm{TP}}{\mathrm{TP}+\mathrm{FP}}$. Low values mean the matcher is confident.
2. **Mutual information:** I(S;Z) between the Bernoulli variable S=1 if both images share the same illumination (diagonal of the grid) and S=0 otherwise. This measures how much a method’s success depends on lighting.
3. **Information-gain rate:** $\mathrm{IG}/t=\left(1-H_{\mathrm{conf}}\right)/\bar{t}$ where $\bar{t}$ is the mean inference time. It rewards methods that deliver lots of useful information per second.

Table 1 summarizes the scores obtained by first summing, for every algorithm, the 30 illumination-condition matrices into one 25×25 match-count matrix and the corresponding elapsed-time arrays into a single vector. From that matrix, true positive (TP) and false positive (FP) counts for each of the 625 image pairs were extracted, converted them to success probabilities $p=\frac{\mathrm{TP}}{\mathrm{TP}+\mathrm{FP}}$, and then applied the three information-theoretic formulas described above: (i) the per-pair Shannon entropy $H_{\mathrm{conf}}$ averaged over all pairs; (ii) the mutual information I(S;Z) between the binary variable *S* that denotes “same illumination” (diagonal of the matrix) and the Bernoulli outcome *Z* of a successful match; and (iii) the information-gain rate $\mathrm{IG}/t=\left(1-H_{\mathrm{conf}}\right)/\bar{t}$, where $\bar{t}$ is the mean inference time computed from the flattened elapsed-time vector.

LightGlue yields both the lowest average uncertainty and the highest information-gain rate (10.6 bits s^−1^), while its illumination dependence is negligible. Although ORB is extremely fast, its high entropy ($H_{\mathrm{conf}}\approx 0.84$) reveals that it is essentially guessing on many pairs. BRISK and AKAZE appear highly sensitive to flash direction, as evidenced by their saturated I(S;Z), whereas SIFT and SuperGlue are slower and less informative overall. Consequently, LightGlue is adopted in the navigation pipeline.

**Figure 4:**
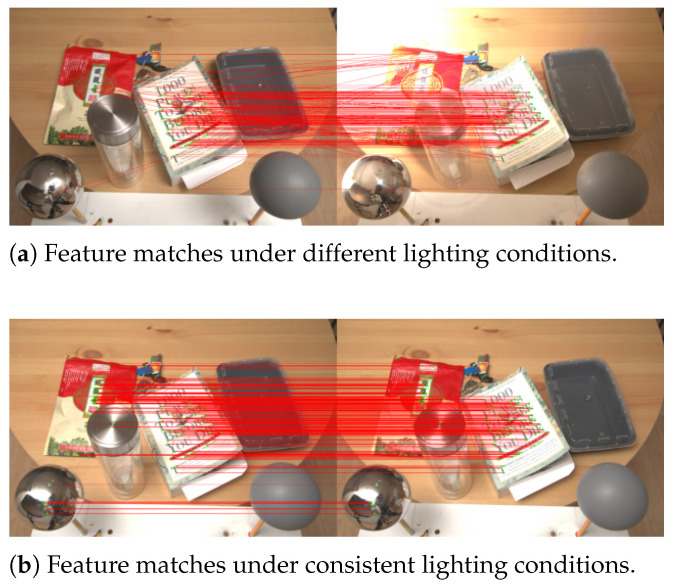
Evaluation of feature matching performance using ORB.

Figure 4 gives a visual impression of the illumination sensitivity that drives the IT scores. Under varying flash positions, ORB produces many spurious correspondences (Figure 4a), whereas same-lighting pairs on the diagonal (Figure 4b) remain reliable.

### 5.2. System Evaluation

This experiment aimed to train and select the navigator, and to deploy it along with the topological map, across four distinct environments within the Habitat simulator: an apartment, Van Gogh room, Skokloster Castle, and a house (identified as 17DRP5sb8fy in the Matterport3D or MP3D dataset [74]). The LightGlue algorithm was employed for feature extraction throughout these tasks (see Section 5.1). It is important to note that one observed frame is acquired at each step or iteration in the simulation and the resolution of every color image and depth map in these experiments is (256, 256) pixels. Furthermore, the simulation parameters corresponding to the agent movement, i.e., $d_{\mathrm{step}}=0.25\;m$ and $\theta_{\mathrm{step}}=10^{\circ }$, are fixed for the entire experimentation.

The model training phase was conducted exclusively in the Skokloster Castle environment, where multiple MLP classifiers were trained and tested from the collected data. The classifier that achieved the highest F-1 score (Equation (19)) during this phase was selected as the navigator, as detailed in Section 4.1. The selected navigator was then utilized in conjunction with a topological map to guide the autonomous navigation process in each of the four environments. Trained models, animations, and codes are provided as Supplementary Materials.

#### 5.2.1. Model Training and Navigator Selection

The model training phase began by collecting RGB-D data by manually controlling the agent through two different paths through the Skokloster Castle, which consisted of 730 and 731 iterations, respectively, as depicted in Section 4.1.1. This process resulted in two groups of observed frames ${\left\{\left(I_{o},D_{o}\right)\right\}}_{1}$ and ${\left\{\left(I_{o},D_{o}\right)\right\}}_{2}$ that served to generate the training visual memories $V_{1}={\left\{\left(I_{\mathrm{key}},D_{\mathrm{key}}\right)\right\}}_{1}$ and $V_{2}={\left\{\left(I_{\mathrm{key}},D_{\mathrm{key}}\right)\right\}}_{2}$, consisting of 94 and 93 keyframes, respectively, as depicted in the visual memory construction process (Section 4.1.2), setting $\epsilon_{\mathrm{select}}=0.9$ which was experimentally defined. It is important to note that the key locations are used for visualization purposes only and are not taken into account by the proposed system. Later, the paths were manually retraced using $V_{1}$ and $V_{2}$ as reference, as depicted in Section 4.1.3, in 868 and 831 iterations, respectively. For this process, two CSV files were generated whose columns included the translation vector t, the rotation in quaternion q, the registration error *e*, and the navigation action *a*. Finally, the files were appended and the dataset D={t,q,e,a}={X,a} resulted in a tensor of shape (1697,9) after removing the two instances of *finish*.

The aggregated dataset D, with a tensor shape of (1697,9), was subsequently divided into training and test sets split in a ratio of 80-20. The distribution of classes within the training set was relatively imbalanced, with 516 instances of *left*, 481 of *forward*, 212 of *right*, and 148 of *update*. The test set maintained a similar distribution pattern, containing 129 instances of *left*, 121 of *forward*, 53 of *right*, and 37 of *update*. To enhance the model’s performance, the features were standardized using the StandardScaler, ensuring that each feature contributed equally to the training process. The scaling parameters were fitted on the training data and subsequently applied to both the training and test sets to maintain consistency.

An MLP classifier was employed to model the navigation actions, with hyperparameters optimized through a grid search approach (Section 4.1.4). The parameter grid encompassed variations in activation functions and solvers with two hidden layer consisting of 100 and 50 units, respectively, in a maximum of 1500 iterations. Specifically, the grid included

- Activation Functions: tanh, relu.
- Solvers: adam, sgd.

The tanh activation function and the sgd solver yielded the highest weighted F-1 score of 0.92, and thus, this trained model, with a total size of 120.2 KB, was selected and implemented as the navigator.

#### 5.2.2. Topological Map and Autonomous Navigation

In this part of the experiment, the capabilities of the system—such as keyframe selection, path planning, obstacle avoidance, and autonomous navigation—were evaluated through eight autonomous navigation tasks covering four different virtual environments.

The system performance is evaluated using the goal distance, defined as the distance between the final key location and the agent’s final location. The latter occurs when *a* = “finish”, as described in Section 4.3.4, and is denoted by (×). The animations corresponding to these tasks are provided as Supplementary Material. The threshold related to the geometric criteria used in the topological map generation (Section 4.2.2) are experimentally fixed to $\epsilon_{\mathrm{dist}}=0.1$ m, $\epsilon_{z}=-0.3$ m, and $\epsilon_{\mathrm{lateral}}=0.5$, through all the navigation tasks. The results of the experiments are summarized in Table 2. During autonomous navigation, the feature-based point cloud registration process (Section 3.2) takes approximately 0.31 ± 0.08 s and the navigator (Section 4.3.4) inference time is approximately 0.721 ± 0.47 milliseconds. Other processes such as localization (Section 4.3.1) and path planning (Section 4.3.2) may vary according to the number of nodes and edges in the Topological Map.

##### Apartment

This scene portrays an urban apartment divided into three distinct areas: a living room furnished with contemporary furniture and plants, an empty hallway that connects the two primary areas through open doors, and a dining room centered around a large table. This environment was chosen to evaluate the system in two path retracing tasks. Unlike generating a topological map representing the environment as a graph, the path retracing tasks consist of following a predefined visual path as a sequence of key locations depicted by keyframes in a visual memory.

**Figure 5:**
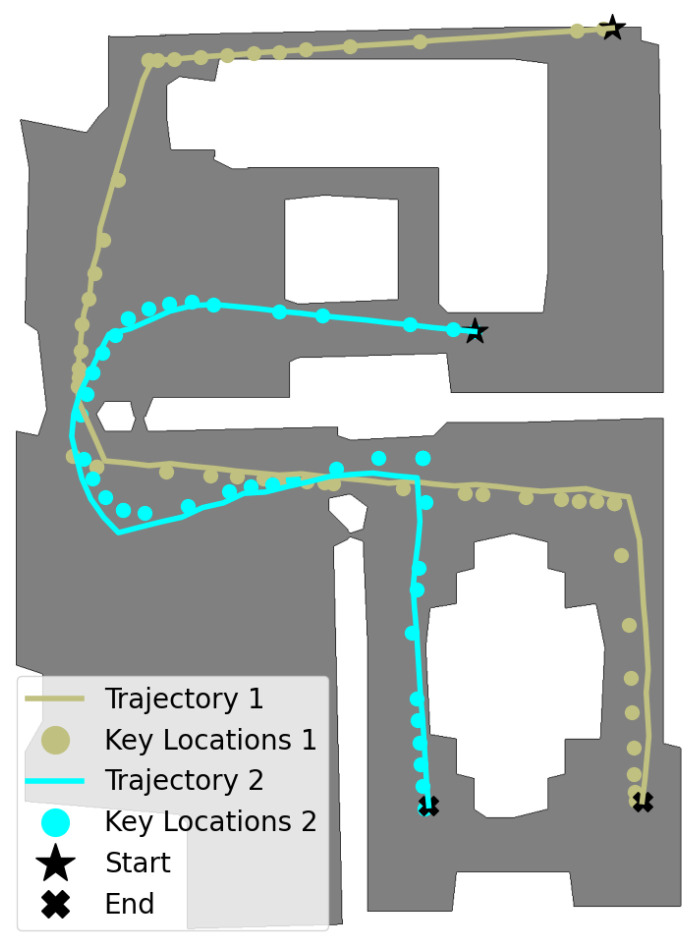
Autonomous navigation tasks conducted in the apartment.

In this experiment, two visual memories, $V_{1}$ and $V_{2}$, were generated, and the navigator was implemented to autonomously follow the visual paths. Specifically, the visual memories were constructed by manually controlling the agent through the environment and selecting consecutive keyframes $\left\{\left(I_{\mathrm{key}},D_{\mathrm{key}}\right)\right\}$ as described in Section 4.1.2. These visual memories were stored as numpy files (.npy), with $V_{1}$ and $V_{2}$ having sizes of 15.7 MB and 21.1 MB, respectively. Then, the navigator, trained as detailed in Section 5.2.1, autonomously retraced both paths using $V_{1}$ and $V_{2}$ as references. The navigation tasks executed in this environment are illustrated in Figure 5.

Task 1 and Task 2: Path Retracing

Initially, the agent was placed at the right side (task-1) and at the left side (task-2) of the living room and manually controlled, through 451 and 92 steps, respectively, to the opposite sides of the dining room. Subsequently, $V_{1}=V_{\mathrm{task}-1}$ and $V_{2}=V_{\mathrm{task}-2}$ were constructed from the observed frames ${\left\{\left(I_{o},D_{o}\right)\right\}}_{1}$ and ${\left\{\left(I_{o},D_{o}\right)\right\}}_{2}$ by setting $\epsilon_{\mathrm{select}}=0.9$, resulting in ${\left|V\right|}_{1}=56$ and ${\left|V\right|}_{2}=46$ keyframes, respectively. This corresponds to selecting one keyframe approximately every eight and two observed frames, respectively.

The autonomous navigation phase began by experimentally defining the thresholds $\epsilon_{\mathrm{visual}}=0.84$ and $\epsilon_{\mathrm{distance}}=0.1\;m$, which trigger the recovery mechanism. Then, the agent was placed at the original initial locations (⋆), and the autonomous navigation began by localizing the agent. The navigator retraced both paths in 223 and 151 steps, covering distances of 45.1 m and 33.29 m, respectively. The distances between the agent final locations and the two final key locations were 0.09 and 0.07 meters.

##### Van Gogh Room

This environment represents the personal bedroom of the renowned artist Vincent van Gogh, meticulously recreated to reflect the late 19th-century aesthetic and spatial layout. It features a confined space with multiple objects placed around the room; therefore, it was considered ideal to evaluate the topological map and autonomous navigation through three tasks: task-3, task-4, and task-5.

During the topological map generation phase, the agent was placed at the left side of the room once and at the right side four times, and manually controlled to the center of the room in 29, 28, 24, 20, and 24 steps, respectively. Five visual memories were then constructed by setting $\epsilon_{\mathrm{select}}=0.94$, resulting in ${\left|V\right|}_{3}=17$, ${\left|V\right|}_{4}=22$, ${\left|V\right|}_{5}=18$, ${\left|V\right|}_{6}=18$, and ${\left|V\right|}_{7}=21$ keyframes. This led to the selection of approximately one or two keyframes for every observed frame.

**Figure 6:**
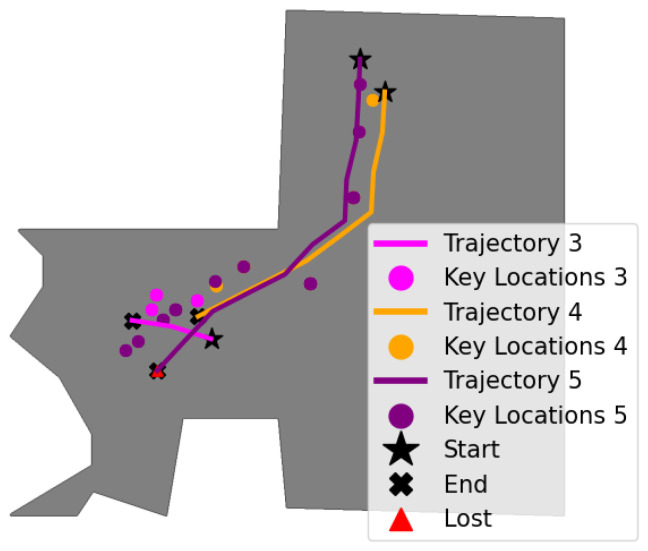
Autonomous navigation tasks conducted in the Van Gogh room.

The topological map was generated by associating each keyframe with a node and connecting them if the visual and geometric criteria were satisfied (see Section 4.2), with thresholds set at $\epsilon_{\mathrm{visual}}=0.94$, $\epsilon_{z}=-0.3\;m$, and $\epsilon_{\mathrm{lateral}}=0.5$. The 96 nodes, stored as pairs of color images and depth maps, had a total size of 44.1 MB and were stored as numpy files (.npy), while the edges, also stored as .npy, had a total size of 73.9 KB. Once the topological map was created, the thresholds that trigger the recovery mechanism were defined as $\epsilon_{\mathrm{visual}}=0.84$ and $\epsilon_{\mathrm{distance}}=0.1\;m$. The autonomous navigation tasks conducted in this environment are illustrated in Figure 6.

Task 3: Short-Range Navigation

The agent started at the center of the room, aiming to reach a keyframe close to it but outside its field of view. The node $N_{94}$ was defined as the end node, and the system began by localizing the agent near node $N_{35}$. The path planning process computed the path $P_{\mathrm{task}-3}=\left(N_{35},N_{37},N_{94}\right)$, resulting in a visual memory composed of the keyframes $V_{\mathrm{task}-3}=\left\{{\left(I_{\mathrm{key}},D_{\mathrm{key}}\right)}_{35},{\left(I_{\mathrm{key}},D_{\mathrm{key}}\right)}_{37},{\left(I_{\mathrm{key}},D_{\mathrm{key}}\right)}_{94}\right\}$. In this task, the agent ended up 0.02 m away from the final key location after traveling a distance of 1.03 meters.

Task 4: Medium-Range Navigation

The agent started next to the bed with the goal of reaching a keyframe (associated with node $N_{52}$) located at the center of the room, requiring a longer traversal than in the previous task. The autonomous navigation phase began by localizing the agent near node $N_{80}$. The computed path was $P_{\mathrm{task}-4}=\left(N_{80},N_{3},N_{4},N_{88},N_{10},N_{52}\right)$, which was converted into a visual memory $V_{\mathrm{task}-4}$. The navigator successfully followed this path in 25 steps, traveling a distance of 4.3 m and concluding the task 0.01 m away from the final key location.

Task 5: Long-Range Navigation with Obstacle Avoidance

The agent started next to the bed, aiming to reach a keyframe (associated with node $N_{39}$) near the table located at the left corner of the room. Initially, the system localized the agent near node $N_{1}$, and the path $P_{\mathrm{task}-5}=\left(N_{1},N_{3},N_{4},N_{88},N_{10},N_{11},N_{14},N_{15},N_{17},N_{18}\right)$ was computed and converted into visual memory $V_{\mathrm{task}-5}$. However, an obstacle was detected when attempting to reach node $N_{18}$, triggering the recovery mechanism (marked with Δ). Consequently, the edge $E_{17,18}$ was disconnected, the agent was re-localized near $N_{18}$, and the path was recomputed as $P_{\mathrm{task}-5}=\left(N_{18}\right)$. The agent then successfully reached this node in two additional steps, completing the task in a total of 40 steps, covering a distance of 5.7 m, and arriving within 0.01 m of the final key location.

##### Skokloster Castle

This virtual replica showcases expansive areas such as grand halls, ornate chambers, extensive corridors, and a central table located in the main hall. The environment maintains consistent lighting conditions and is rich in textures. However, it features repetitive patterns in the flooring and similar content on opposing walls, which can pose unique challenges for feature detection and matching algorithms. The castle was chosen to evaluate the system through two tasks: task-6 and task-7.

During the topological map generation phase, the agent was manually controlled across four paths consisting of 323, 315, 325, and 362 steps, covering most parts of the castle. Four visual memories were then constructed by setting $\epsilon_{\mathrm{select}}=0.95$, resulting in ${\left|V\right|}_{8}=33$, ${\left|V\right|}_{9}=36$, ${\left|V\right|}_{10}=34$, and ${\left|V\right|}_{11}=28$ keyframes. This corresponds to selecting approximately one keyframe every ten to thirteen observed frames.

**Figure 7:**
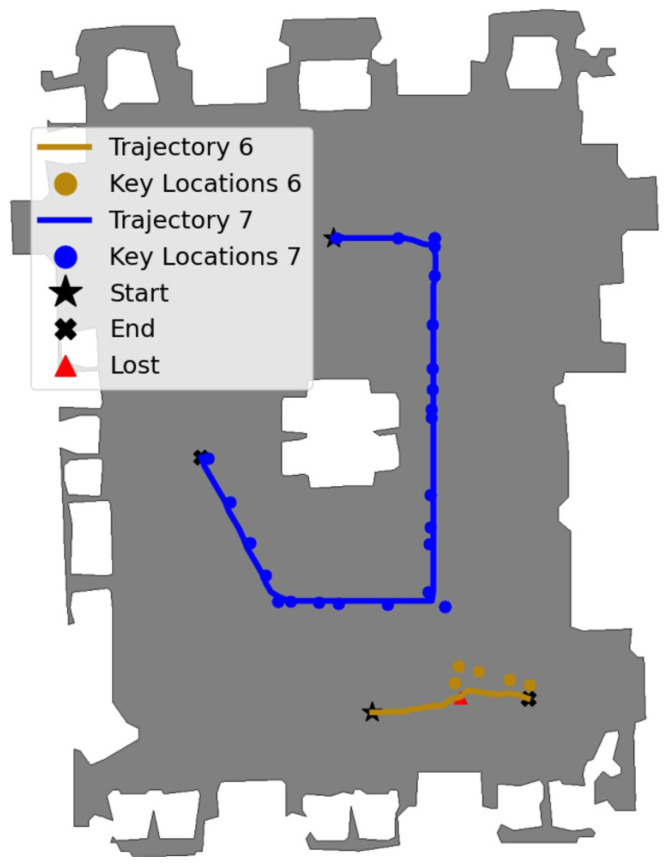
Autonomous navigation tasks conducted in the Skokloster castle.

The keyframes composing the visual memories were associated with nodes in the topological map and connected if the visual and geometric criteria were satisfied, with thresholds set at $\epsilon_{\mathrm{visual}}=0.95$, $\epsilon_{z}=-0.3\;m$, and $\epsilon_{\mathrm{lateral}}=0.5$. The 131 nodes, stored as pairs of color images and depth maps, occupied 60.1 MB as numpy files (.npy), while the edges, also stored as .npy, required 137.4 KB or memory space. The thresholds that trigger the recovery mechanism were defined as $\epsilon_{\mathrm{visual}}=0.84$ and $\epsilon_{\mathrm{distance}}=0.1\;m$. The two next tasks are illustrated in Figure 7 and detailed below.

Task 6: Long-Distance Navigation with Path Replanning

This task involved traversing the castle from one door to another located at opposite sides, combining key locations from two different paths traversed during the topological map generation phase. Initially, the final node was set to $N_{31}$, and the agent was localized near node $N_{75}$ (⋆). The system computed the path $P_{\mathrm{task}-6}=\left(N_{75},N_{74},N_{28},N_{29},N_{30},N_{31}\right)$, and the navigator attempted to follow it. The agent successfully reached the first four nodes, but a low similarity score $s=0.82<\epsilon_{\mathrm{visual}}$ was attained when attempting to reach $N_{30}$, triggering the recovery mechanism (Δ). The edge $E_{29,30}$ was disconnected, and the path was recomputed from the current node $N_{29}$ to the end node $N_{31}$, resulting in $P_{\mathrm{task}-6}=\left(N_{29},N_{79},N_{77},N_{76},N_{75},N_{74},N_{28},N_{29},N_{30},N_{31}\right)$. The agent successfully followed the new path, covering a distance of 11.1 m and completing the task in a total of 60 steps, ultimately stopping 0.21 m from the final key location.

Task 7: Extended Navigation

This task involved navigating through the castle by following an elliptical path around the central table, maintaining a consistent distance between the walls and the table, in a clockwise direction. The same values for the thresholds used in the previous task were used in this task as well. The final node was defined as $N_{61}$, and the system localized the agent near $N_{0}$ (⋆). The path computed was $P_{\mathrm{task}-7}=\left(N_{0},N_{1},N_{3-14},N_{16-19},N_{39-40},N_{42-54},N_{56},N_{58-61}\right)$, composed of 38 nodes. The visual memory $V_{\mathrm{task}-7}$ was directly generated from $P_{\mathrm{task}-7}$. The navigator successfully conducted the agent through the entire path, covering a larger distance of 50.43 m, successfully concluding the task in 167 steps and ending up within 0.15 m of the final key location.

##### MP3D House

This virtual environment represents a common indoor space with multiple rooms, including one bedroom, two bathrooms, one toilet, a TV room, a dining room, an entryway, a living room, a family room or lounge, and a kitchen (The MP3D house can be visualized in: https://aspis.cmpt.sfu.ca/scene-toolkit/scans/house-viewer?condition=mpr3d&modelId=mp3d.17DRP5sb8fy) (visited on 1 May 2025). The environment features various pieces of furniture and household items, providing a rich set of visual textures and spatial configurations. It was chosen because it represents a typical indoor setting with multiple interconnected rooms, allowing the evaluation of the system’s navigation capabilities in a complex but common scenario. The environment was used to evaluate the system through task-8.

During the topological map generation phase, the agent was manually controlled across two paths consisting of 79 and 71 steps, respectively. The first path went from the family room to the dining room, traversing the bedroom and living room. The second path went from the bedroom to the entryway, passing through the living room and dining room. Two visual memories were then constructed by setting $\epsilon_{\mathrm{select}}=0.94$, resulting in ${\left|V\right|}_{12}=62$ and ${\left|V\right|}_{13}=54$ keyframes. This corresponds to selecting approximately one keyframe every one to two observed frames.

The keyframes composing the visual memories were associated with nodes in the topological map and connected if the visual and geometric criteria were satisfied, with thresholds set at $\epsilon_{\mathrm{visual}}=0.88$, $\epsilon_{z}=-0.3\;m$, and $\epsilon_{\mathrm{lateral}}=0.5$. The 116 nodes, stored as pairs of color images and depth maps, occupied 53.2 MB as numpy files (.npy). The threshold that triggers the recovery mechanism for distance was defined as $\epsilon_{\mathrm{distance}}=0.1\;m$, and the similarity threshold was experimentally set to $\epsilon_{\mathrm{visual}}=0.83$.

Task 8: Traversing the House Across the Main Corridor

**Figure 8:**
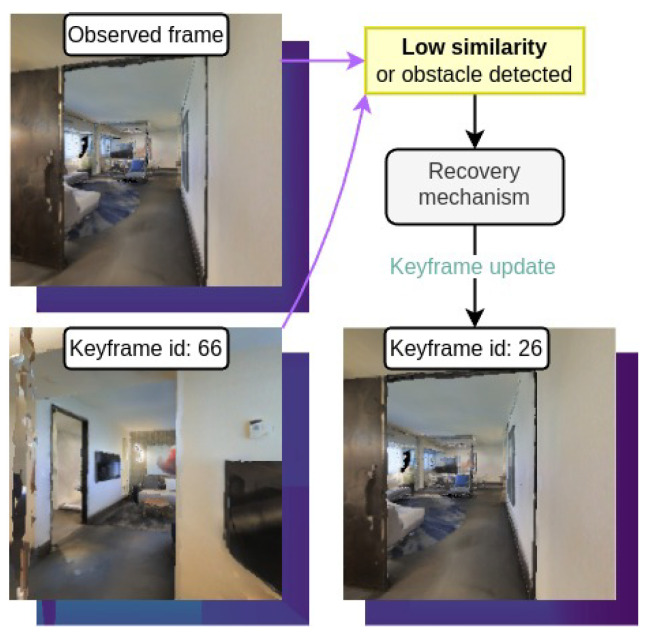
Recovery mechanism correcting the agent’s path after low similarity detection.

This task involved navigating the house through the main corridor that connects multiple rooms, requiring the computation of a path that combines both visual memories $V_{12}$ and $V_{13}$. The agent was manually placed in the family room and aimed to reach node $N_{117}$. The autonomous navigation began by correctly localizing the agent close to node $N_{16}$ and then computing the path $P\mathrm{task}-8=(N16,N_{23},N_{65},N_{66},N_{69},N_{116},N_{117})$. The recovery mechanism was triggered four times, at steps: 16, 69, 71, and 74. Particularly, from steps 13 to 15, the agent incorrectly attempted to reach nodes $N_{65}$ and $N_{66}$ (as shown in Figure 8), but the recovery mechanism was triggered at step 16 due to a low similarity score (see Section 4.3.3). The edge $E_{23,65}$ was disconnected, and the path was corrected by assigning $N_{26}$ as the next keyframe.

**Figure 9:**
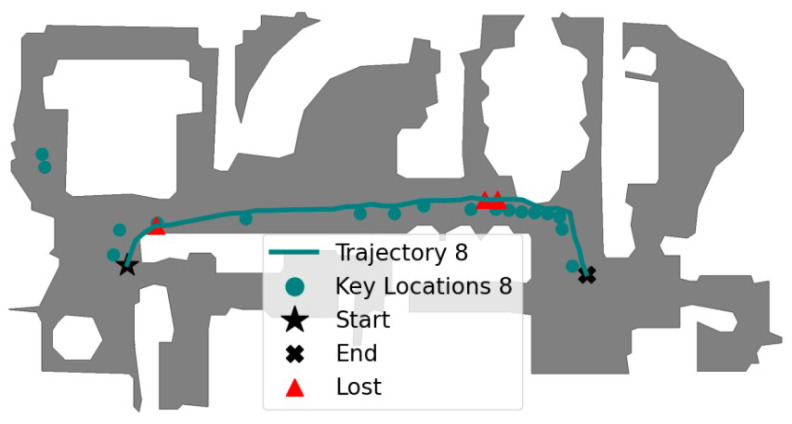
Autonomous navigation task conducted in the house.

The final path followed by the agent in 114 steps was $P_{\mathrm{task}-8}=(N_{26},N_{31},N_{38},N_{94},N_{43},N_{101},N_{104-106},N_{113},$$N_{107-109},N_{115},$$N_{108},N_{110},N_{111},N_{114},N_{117})$. After traveling 39.5 m, the agent ended up 0.28 m away from the final key location, mainly due to its the kinematic constraints. This task is illustrated in Figure 9.

## 6. Discussion

The proposed modular, vision-based navigation system demonstrates the feasibility of using RGB-D data and a neural network-based navigator for autonomous navigation tasks in indoor environments. By leveraging a topological map composed of interconnected keyframes and simplifying the decision-making process, the system effectively integrates conventional vision-based navigation with end-to-end learning approaches. Notably, a single MLP trained only on the Castle scene achieved successful navigation in all four environments, highlighting strong out-of-domain generalization. Future work will consider transfer learning or reinforcement learning if new modules are introduced to deal with more challenging environments.

Compared to traditional SLAM methods, which often require exhaustive exploration and precise metric representations [75], the proposed system significantly reduces computational resources by representing the environment through a graph of interconnected keyframes (Section 4.2). This approach eliminates the need for complete environmental representations and avoids the accumulation of errors from positional sensors such as odometry or GPS. By focusing on navigable paths rather than detailed maps, the system addresses the loop closure problem inherent in SLAM and reduces memory requirements.

The information-theoretic evaluation clarifies why LightGlue, rather than merely performing well geometrically, offers the most suitable trade-off for the navigation task. Its low $H_{\mathrm{conf}}$ indicates that, across the full illumination grid, LightGlue’s match decisions carry little internal uncertainty, while the very small I(S;Z) shows that this confidence is essentially lighting-invariant. ORB’s high information-gain rate is driven almost entirely by its micro-second runtime; the large entropy value ($H_{\mathrm{conf}}\approx 0.84$) exposes that many of those rapid decisions are near random and therefore propagate noise into the relative-pose estimator. BRISK and AKAZE achieve impressively low entropy, yet their saturated I(S;Z) scores reveal a strong, highly undesirable coupling between success and the exact flash position—an artifact that would translate into brittle behaviour under real-world lighting changes. The IT metrics therefore corroborate the empirical navigation results: LightGlue maximizes useful pose information per second without introducing environment-specific biases, explaining its consistent advantage when the system is deployed in previously unseen scenes. According to [76], LightGlue remains faster than OmniGlue. OmniGlue’s foundation-model guidance, however, promises greater robustness on unseen domains and may be preferred when that priority outweighs strict real-time constraints.

The MLP-based navigator, trained as a classifier on relative pose estimations and kinematic constraints, proved effective in predicting navigation actions. Despite being trained on a small dataset collected from a single environment, the navigator was successfully deployed across four simulated environments without additional training. This generalization capability suggests that the decision-making process, simplified through relative pose estimation and imitation learning, is robust to changes in environmental contexts. In contrast, end-to-end methods and reinforcement learning approaches typically require large datasets and intensive training phases for each new environment [77]. For instance, the dataset used in [78] for training and evaluating their CNN-based navigation system using an imitation learning approach comprised 14,412 color images at a resolution of (320, 160) pixels, occupying approximately 2.11 GB in storage. In comparison, the navigator was trained using two CSV files (Section 5.2.1) (The relevant columns of the CSV files were: x, y, z, q_0_, q_1_, q_2_, q_3_, rmse, and action), one file per manual path retracing as explained in Section 4.1.3. These files consisted of 1697 rows and 10 columns: the translation vector t=(x,y,z) as in Equation (13), the rotation (Equation (12)) expressed in quaternion form $q=(q_{0},q_{1},q_{2},q_{3})$, the registration error *e* as in Equation (14), and a reference visual memory index that was not utilized during training. The total memory footprint of these files was 303.7 KB, representing approximately 0.01% (10,000 times less) of the dataset size used in [78]. Despite being a significantly smaller dataset, the navigator, trained in one environment, was able to guide the agent through the four environments while preserving a mean goal error of 0.10 m (Section 5.2.2).

The system extensively employs image similarity for generating visual memories, constructing the topological map, and triggering the recovery mechanism (Section 4). To compute image similarity, feature embeddings are extracted from color images (Section 3.1). Vision Transformers (ViT) were considered for this task due to their advantages in modeling long-range dependencies and adaptability to different input sizes [79]. However, ViTs involve higher computational complexity and face scalability challenges, which conflict with the cost-effectiveness aspect of the proposed system. Therefore, CNNs were preferred because they offer lower computational demands and have a proven track record of effectiveness. Importantly, the modular design of our system allows for the future integration of ViTs as needed, accommodating evolving requirements and advancements in deep learning technologies. Empirically, setting the low-similarity threshold to $\epsilon_{\mathrm{nav}}\;\approx \;0.84$ enabled successful navigation across all tasks, while the keyframe density stayed consistent for $\epsilon_{\mathrm{select}}\in \left[0.90,0.95\right]$ (Section 5.2.2). Introducing an *adaptive* visual-link threshold—e.g., via cosine-distance clustering [80]—could automatically tune $\epsilon_{\mathrm{visual}}$ and thus preserve robust node connectivity while avoiding manual tuning. Furthermore, methods like Viewpoint-Invariant Adversarial Training (VIAT) [81] could enhance the robustness of ResNet50 to viewpoint changes when computing visual similarity, while ResNet50 combined with AugMix [82] could help reduce sensitivity to illumination variations [83] when the system gets implemented in real environments.

The use of the Habitat simulated environment facilitated the evaluation process by providing built-in tools for easy implementation and testing of the navigation system (Section 5.2). Its provision of direct global positioning data simplified development and allowed for accurate assessment of the system’s performance in realistic virtual indoor settings. When deploying the system in real environments, images with poor sharpness (detected via the robust blur metric of [84]) plan to be skipped, mitigating motion-blur failures without extra computation. When blur is unavoidable (e.g., during fast turns) a lightweight DBLRNet [85] module can be employed to deblur images during autonomous navigation, while low-illuminance frames can be enhanced by MB-TaylorFormer [86] before feature extraction. Furthermore, Gridformer [87] is consider to be used in outdoor environment for image restoration under adverse weather conditions which can potentially affect some key processes, such as localization and recovery mechanism (Section 4.3).

However, the system does present certain limitations. Latency profiling shows feature registration 0.31 ± 0.08 s and navigation inference 0.72 ± 0.47 ms; LightGlue’s architecture enables users to balance the performance-speed ratio to match the embedded computer limitations. Setting thresholds such as $\epsilon_{\mathrm{visual}}$ and $\epsilon_{\mathrm{visual}}$ proved challenging, as they vary depending on the environment. Additionally, the navigator was not trained to perform complex maneuvers requiring sequential actions to avoid obstacles. Incorporating architectures capable of handling temporal sequences, such as Long Short-Term Memory (LSTM) networks [88], could address this limitation in future work. For kinesthetic realism, the action set can be extended with “forward-slow” and arc motions that map directly to wheel velocities, retaining discrete learning while approximating continuous control.

The experiments were conducted in static, simulated environments with consistent illumination, which does not fully reflect the variability encountered in real-world settings. Dynamic environments with moving obstacles or varying lighting conditions were not tested and represent an area for future exploration. Implementing the system on real hardware will introduce additional challenges, including handling computation flow, sensor communication, and environmental changes such as illumination variations and dynamic objects. Future work will port the pipeline to a real RGB-D platform and couple the existing recovery mechanism with a dynamic recognition module [89] that detects and avoids pedestrians without triggering the recovery mechanism, thus maintaining the topological map connections (Section 4.3). Moreover, other metrics such as navigation success, goal-error statistics, power consumption, and control loop frequency will measure the system performance in real implementation.

The density of the topological map impacts the system’s reliability. A practical extension is to monitor edge-failure frequency and automatically spawn additional keyframes around any chokepoint (as in [90]), incrementally densifying the graph whenever sparse connectivity threatens recovery. Nonetheless, this approach will require a more intensive mapping phase (Section 4.2). Complementarily, an age-based pruning policy, powered by semantic segmentation [91], could retire obsolete nodes and edges, allowing the map to evolve with furniture rearrangements or other long-term changes. The system’s efficiency is demonstrated by its traversal of 190.44 m across eight navigation tasks in the Habitat environment, achieving an average goal distance of approximately 0.1 m per task, despite relying on low-resolution data and a limited number of keyframes. However, sparse maps may limit the recovery mechanism’s effectiveness if the agent cannot replan a path or if movement constraints prevent certain maneuvers.

In terms of computational efficiency, the system showed potential for real-time application. Particularly, the relative pose estimation combined with the navigator’s prediction also contributes to computational load but did not impede the system’s performance in the conducted experiments (Section 5.2.2). This efficiency offers practical benefits, such as enabling the use of lower-power hardware, reducing both cost and energy consumption. By reducing reliance on expensive sensors like LiDAR and heavy computational resources, the system potentially becomes more accessible and appealing for broader societal applications. Additionally, efficient resource usage extends battery life and enhances adaptability across various robotic platforms [92].

Future work includes deploying the system on real hardware to assess its performance in dynamic and variable environments. Enhancements to the localization process, such as integrating feature embeddings and cosine similarity, could improve robustness. Transitioning to RGB cameras with depth estimation through deep learning techniques, e.g., Marigold [93], may reduce hardware requirements. Moreover, if the depth channel becomes unreliable (e.g., at long range or on reflective glass) frames whose registration error exceeds a threshold could be discarded and the recovery module temporarily would switch to RGB-only localization. Depth is re-introduced only when confidence improves, with missing values filled by Marigold predictions, so localization remains continuous even under severe depth noise. Incorporating additional modules for user interaction, object recognition, obstacle avoidance, and exploiting depth maps more effectively are also potential avenues for improvement. Additionally, we plan to implement adaptive thresholds jointly with visual features, replacing hand-tuned ϵ values by confidence-aware predictors.

Overall, the system demonstrates a promising approach to autonomous navigation using RGB-D data and a modular design. Its ability to generalize across different environments without retraining suggests that it could be a practical solution for indoor navigation tasks, provided that future work addresses the identified limitations and challenges.

## 7. Conclusions

This paper introduced a modular, vision-based navigation system that leverages RGB-D data and a neural network-based navigator for autonomous navigation in indoor environments. By constructing a topological map composed of interconnected keyframes and simplifying the decision-making process to relative pose estimation and kinematic constraints, the system navigates effectively without requiring exhaustive environmental mapping or extensive training data.

An information-theoretic evaluation—using confidence entropy, mutual information, and information-gain rate—was employed to choose LightGlue as the feature algorithm, ensuring that the visual approach uses a reliable and time-efficient algorithm. The system demonstrated the ability to generalize across different simulated environments without retraining the navigator, indicating its potential applicability for practical indoor navigation tasks. The use of a simple MLP classifier, trained on a small dataset from a single environment, contrasts with other methods that depend on large datasets and intensive training phases. The modular design also allows for scalability and the future integration of additional modules, such as user interaction or object recognition.

Despite its promising performance, the system has limitations that warrant further investigation. The need to manually set thresholds for keyframe connections and visual similarity can affect performance across different environments. Additionally, the navigator was not trained to handle complex maneuvers requiring sequential actions, limiting its effectiveness in dynamic or more complex settings. Incorporating advanced architectures like recurrent neural networks and testing in real-world environments with dynamic elements are important next steps.

Future work will focus on deploying the system on real hardware to evaluate its performance under variable conditions, including dynamic obstacles and changing illumination. Enhancements to the localization process, such as integrating feature embeddings and cosine similarity measures, may improve robustness. Exploring the use of RGB cameras with depth estimation techniques could reduce hardware requirements and increase applicability.

In conclusion, the proposed system offers a viable approach to autonomous indoor navigation using RGB-D data within a modular framework. By reducing hardware complexity and simplifying training requirements, it provides a foundation for developing reliable and cost-effective navigation solutions suitable for a range of indoor environments.

## Figures and Tables

**Table 1:** Information-theoretic comparison of feature algorithms.

Algorithm	$H_{\mathrm{conf}}\;↓$	I(S;Z)↓	IG/t↑
LightGlue	0.081	0.0123	**10.62**
ORB	0.842	0.1589	8.04
BRISK	0.020	2.0293	6.45
AKAZE	0.012	1.0881	4.72
SIFT	0.086	0.3411	2.55
SuperGlue	0.193	0.0119	2.01

**Table 2:** Summary of autonomous navigation tasks and results.

task	Env.	$\epsilon_{\mathrm{select}}$	$\epsilon_{\mathrm{visual}}$	$\epsilon_{\mathrm{nav}}$	Manual	Key-	Auto	Recov.	Goal	Traj.
					Steps	Frames	Steps	Mech.	Dist. (m)	Len. (m)
1	Apart.	0.9	N/A	0.84	451	56	223	No	0.09	45.1
2	Apart.	0.9	N/A	0.84	92	46	151	No	0.07	33.3
3	Room	0.94	0.94	0.84	105	3	11	No	0.02	1
4	Room	0.94	0.94	0.84	105	6	25	No	0.01	4.3
5	Room	0.94	0.94	0.84	105	10	40	Obs. det.	0.01	5.7
6	Castle	0.95	0.95	0.84	1325	13	60	Low sim.	0.21	11.1
7	Castle	0.95	0.95	0.84	1325	38	167	No	0.15	50.4
8	House	0.94	0.88	0.83	150	23	113	Low sim.	0.28	39.5

## Data Availability

Data and code will be available through a GitHub repository if accepted.

## Supplementary Materials

The following supporting information can be downloaded at: https://www.mdpi.com/article/10.3390/e27060641/s1. Video S1: Animated navigation tasks.

## Appendix A. Algorithms

**Algorithm A1** Estimate Relative Pose Using RGB-D Data and Point Clouds
Require: Source color image $I_{o}$, source depth map $D_{o}$, target color image $I_{\mathrm{key}}$, target depth map $D_{\mathrm{key}}$, intrinsic camera parameters matrix K Ensure: Estimated rotation matrix *R*, translation vector t, registration error *e* 1: Detect and match keypoints in $I_{o}$ and $I_{\mathrm{key}}$ using a point feature algorithm. 2: Convert image coordinates (u,v) to normalized image coordinates (x,y) using the inverse of K: $x=\left(u-c_{x}\right)/f_{x}$ $y=\left(v-c_{y}\right)/f_{y}$ 3: For each matched keypoint, retrieve depth value *Z* from $D_{o}$ or $D_{\mathrm{key}}$ 4: Project keypoints to 3D coordinates: X=Z·x Y=Z·y 5: Generate source and target indexed point clouds $P_{o}$ and $P_{\mathrm{key}}$. 6: Compute centroids of corresponding points in both point clouds: ${\bar{P}}_{o}=\frac{1}{n}\sum P_{o_{i}}$ ${\bar{P}}_{\mathrm{key}}=\frac{1}{n}\sum P_{{\mathrm{key}}_{i}}$ 7: Compute the cross-covariance matrix *H* and perform SVD: $H=\sum \left(P_{o_{i}}-{\bar{P}}_{o}\right){\left(P_{{\mathrm{key}}_{i}}-{\bar{P}}_{\mathrm{key}}\right)}^{⊤}$ $U\Sigma V^{⊤}=\mathrm{SVD}\left(H\right)$ 8: Calculate the rotation matrix *R* and translation vector t: $R=VU^{⊤}$ $t={\bar{P}}_{\mathrm{key}}-R{\bar{P}}_{o}$ 9: Apply the transformation and compute the RMSE: $e=\sqrt{\frac{1}{n}\sum {∥\left(RP_{o_{i}}+t\right)-P_{{\mathrm{key}}_{i}}∥}^{2}}$ 10: Convert rotation matrix *R* to quaternion q: R→q Ensure: Estimated rotation matrix *R*, translation vector t, registration error *e*, rotation quaternion q

**Algorithm A2** Visual Memory Construction Algorithm During Model Training
Require: Sequence of observed frames ${\left\{\left(I_{o_{i}},D_{o_{i}}\right)\right\}}_{i=1}^{N}$; Similarity threshold $\epsilon_{\mathrm{select}}$ Ensure: Visual memory V containing selected keyframes 1: Initialize visual memory: V←∅ 2: Compute embedding of the first frame: $e_{\mathrm{key}}\leftarrow \mathrm{ResNet}50\left(I_{o_{1}}\right)$ 3: Set initial keyframe: $\left(I_{\mathrm{key}},D_{\mathrm{key}}\right)\leftarrow \left(I_{o_{1}},D_{o_{1}}\right)$ 4: Add initial keyframe to visual memory: $V\leftarrow V\cup \{\left(I_{\mathrm{key}},D_{\mathrm{key}}\right)\}$ 5: **for** i←2 to *N* **do** 6: Compute embedding of current observed frame: $e_{o_{i}}\leftarrow \mathrm{ResNet}50\left(I_{o_{i}}\right)$ 7: Compute similarity: $s\leftarrow \mathrm{sim}(e_{\mathrm{key}},e_{o_{i}})$ 8: **if** $s<\epsilon_{\mathrm{select}}$ **then** 9: Update keyframe: $\left(I_{\mathrm{key}},D_{\mathrm{key}}\right)\leftarrow \left(I_{o_{i-1}},D_{o_{i-1}}\right)$ 10: Update keyframe embedding: $e_{\mathrm{key}}\leftarrow e_{o_{i}}$ 11: Add new keyframe to visual memory: $V\leftarrow V\cup \{\left(I_{\mathrm{key}},D_{\mathrm{key}}\right)\}$ 12: **end if** 13: **end for** 14: **if** $(I_{o_{N}},D_{o_{N}})\notin V$ **then** 15: Add last frame to visual memory: $V\leftarrow V\cup \{\left(I_{o_{N}},D_{o_{N}}\right)\}$ 16: **end if**

**Algorithm A3** Topological Map Generation Algorithm
Require: Set of keyframes ${\left\{\left(I_{\mathrm{key}},D_{\mathrm{key}}\right)\right\}}_{\mathrm{key}=1}^{N}$, Visual similarity threshold $\epsilon_{\mathrm{visual}}$, Forward movement threshold $\epsilon_{z}$, Lateral displacement ratio threshold $\epsilon_{\mathrm{lateral}}$ Ensure: Topological map G=(N,E) 1: Initialize node set: $N\leftarrow \{N_{\mathrm{key}}=\left(I_{\mathrm{key}},D_{\mathrm{key}}\right)|k=1,\ldots ,N\}$ 2: Initialize edge set: E←∅ 3: **for** k←1 to *N* **do** 4: Compute embedding: $e_{\mathrm{key}}\leftarrow \mathrm{ResNet}50\left(I_{\mathrm{key}}\right)$ 5: **end for** 6: **for** each ordered pair $\left(N_{i},N_{j}\right)$ where i≠j **do** 7: Compute cosine similarity: $s_{i,j}\leftarrow \mathrm{sim}\left(e_{i},e_{j}\right)$ 8: **if** $s_{i,j}>\epsilon_{\mathrm{visual}}$ **then** ▹ Connection criterion satisfied 9: Compute transformation $\left(R_{i,j},t_{i,j}\right)$ between $\left(I_{i},D_{i}\right)$ and $\left(I_{j},D_{j}\right)$ 10: Extract translation components: $t_{x}$, $t_{y}$, $t_{z}$ from $t_{i,j}$ 11: Compute lateral displacement: $d_{\mathrm{lateral}}\leftarrow \sqrt{t_{x}^{2}+t_{y}^{2}}$ 12: Compute ratio: $r\leftarrow \frac{d_{\mathrm{lateral}}}{t_{z}}$ 13: **if** $t_{z}>\epsilon_{z}$ **then** ▹ Backward constraint satisfied 14: **if** $r<\epsilon_{\mathrm{lateral}}$ **then** ▹ Lateral displacement constraint satisfied 15: Add edge: $E_{i,j}\leftarrow \left(N_{i},N_{j}\right)$ 16: Update edge set: $E\leftarrow E\cup \{E_{i,j}\}$ 17: **end if** 18: **end if** 19: **end if** 20: **end for** 21: **return** Topological map: G=(N,E)

**Algorithm A4** Localization and Initial Positioning Algorithm
Require: Current RGB-D observation $\left(I_{o},D_{o}\right)$, Set of keyframes ${\left\{\left(I_{\mathrm{key}},D_{\mathrm{key}}\right)\right\}}_{\mathrm{key}=1}^{N}$, Translation step size $d_{\mathrm{step}}$, Rotation step size $\theta_{\mathrm{step}}$ Ensure: Initial node $N_{\mathrm{current}}$ in the topological map 1: **Initialization** 2: Initialize cost list C←∅ 3: **for** k←1 to *N* **do** 4: Estimate $\left(R_{o,\mathrm{key}},t_{o,\mathrm{key}}\right)\leftarrow \mathrm{RelativePoseEstimation}\left(\left(I_{o},D_{o}\right),\left(I_{\mathrm{key}},D_{\mathrm{key}}\right)\right)$ 5: Extract forward translation component $t_{z}\leftarrow t_{o,\mathrm{key}}\left[z\right]$ 6: **if** $t_{z}>0$ **then** ▹ Keyframe is frontal 7: Compute translation magnitude $|t_{o,\mathrm{key}}|$ 8: Extract yaw angle ϕ from rotation matrix $R_{o,\mathrm{key}}$ 9: Compute cost: $$C_{\mathrm{key}}\leftarrow \left\lceil \frac{|t_{o,\mathrm{key}}|}{d_{\mathrm{step}}}\right\rceil +\left\lceil \frac{\left|\phi \right|}{\theta_{\mathrm{step}}}\right\rceil$$ 10: Add $\left(k,C_{\mathrm{key}}\right)$ to cost list C 11: **end if** 12: **end for** 13: **if** C is not empty **then** 14: Find minimal cost $C_{\min}\leftarrow \min\left\{C_{\mathrm{key}}|\left(k,C_{\mathrm{key}}\right)\in C\right\}$ 15: Collect keyframes with minimal cost: $$K_{\min}\leftarrow \left\{k|\left(k,C_{\mathrm{key}}\right)\in C,\;C_{\mathrm{key}}=C_{\min}\right\}$$ 16: Select keyframe with lowest index: $$k_{\mathrm{initial}}\leftarrow \min\left\{k|k\in K_{\min}\right\}$$ 17: Set initial node $N_{\mathrm{current}}\leftarrow N_{k_{\mathrm{initial}}}$ 18: **else** 19: **Error:** No frontal keyframes found. Cannot localize agent. 20: **end if** 21: **return** Initial node $N_{\mathrm{current}}$

**Algorithm A5** Autonomous Navigation Algorithm
Require: Topological map G=(N,E), Trained MLP navigator, Current RGB-D observation $\left(I_{o},D_{o}\right)$, Goal node $N_{\mathrm{goal}}$ Ensure: Successful navigation from current location to $N_{\mathrm{goal}}$ 1: Compute embeddings for keyframes: $e_{\mathrm{key}}\leftarrow \mathrm{ResNet}50\left(I_{\mathrm{key}}\right)$ for each $N_{\mathrm{key}}\in N$ 2: Localize agent to initial node $N_{\mathrm{current}}$ using Algorithm 4 3: Compute shortest path $P\leftarrow \mathrm{Dijkstra}(G,N_{\mathrm{current}},N_{\mathrm{goal}})$ 4: Generate visual memory V← sequence of keyframes along P 5: **while** $N_{\mathrm{current}}\neq N_{\mathrm{goal}}$ **do** 6: Capture current RGB-D observation $\left(I_{o},D_{o}\right)$ 7: Estimate $\left(t_{i},q_{i},e_{i}\right)\leftarrow \mathrm{RelativePoseEstimation}\left(\left(I_{o},D_{o}\right),N_{\mathrm{current}}\right)$ 8: Suggest action $a_{i}\leftarrow \mathrm{Navigator}\left(t_{i},q_{i},e_{i}\right)$ 9: **if** $\mathrm{ObstacleDetected}(D_{o},a_{i})$ **then** 10: Remove edge $E_{\mathrm{current},\;\mathrm{next}}$ from *G* 11: Recompute path $P\leftarrow \mathrm{Dijkstra}(G,N_{\mathrm{current}},N_{\mathrm{goal}})$ 12: Update visual memory V 13: **Continue** 14: **end if** 15: Compute similarity $s_{i}\leftarrow \mathrm{sim}\left(\mathrm{ResNet}50\left(I_{o}\right),e_{\mathrm{current}}\right)$ 16: **if** $s_{i}<\epsilon_{\mathrm{visual}}$ **then** 17: Remove edge $E_{\mathrm{current},\;\mathrm{next}}$ from *G* 18: Recompute path $P\leftarrow \mathrm{Dijkstra}(G,N_{\mathrm{current}},N_{\mathrm{goal}})$ 19: Update visual memory V 20: **Continue** 21: **end if** 22: Execute action $a_{i}$ 23: **if** $a_{i}$ is “*update memory*” **then** 24: **if** $N_{\mathrm{current}}\neq N_{\mathrm{goal}}$ **then** 25: Update $N_{\mathrm{current}}$ to next node in P 26: **else** 27: **Break** 28: **end if** 29: **end if** 30: **end while** 31: **return** Successful navigation to $N_{\mathrm{goal}}$
