# Supplementary figures and images for: Efficient Learning-Based Robotic Navigation Using Feature-Based RGB-D Pose Estimation and Topological Maps
Source: Entropy (Basel). 2025 Jun 15;27(6):641. doi: 10.3390/e27060641 (PMC12191688; doi:10.3390/e27060641)

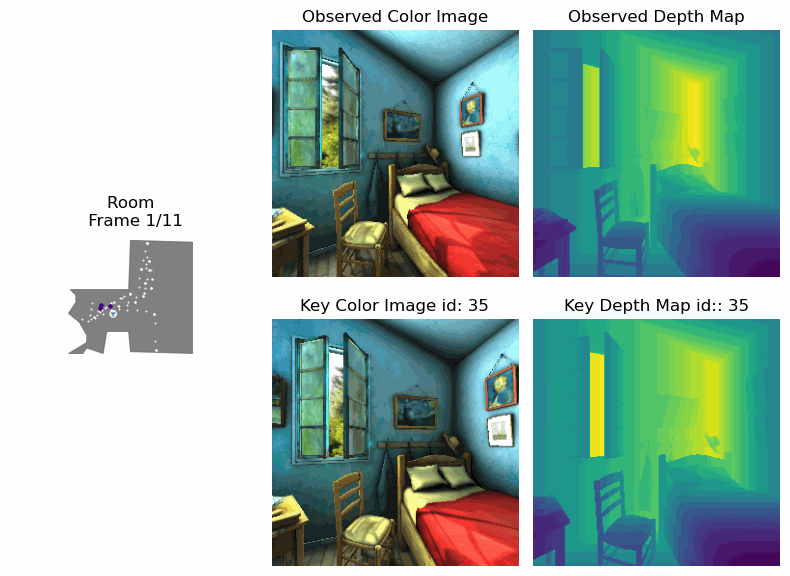

Supplement: Supplementary file 1 [file entropy-27-00641-s001.zip › supplementary_material/task-3.gif]

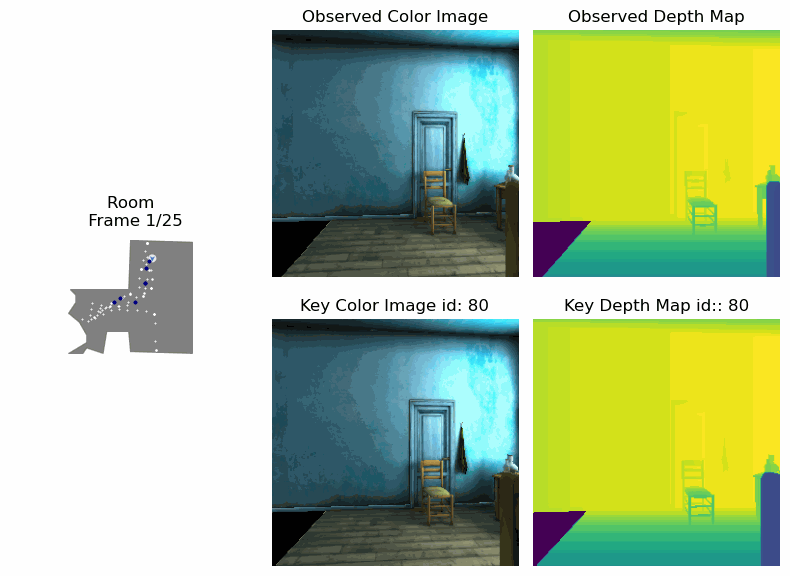

Supplement: Supplementary file 1 [file entropy-27-00641-s001.zip › supplementary_material/task-4.gif]

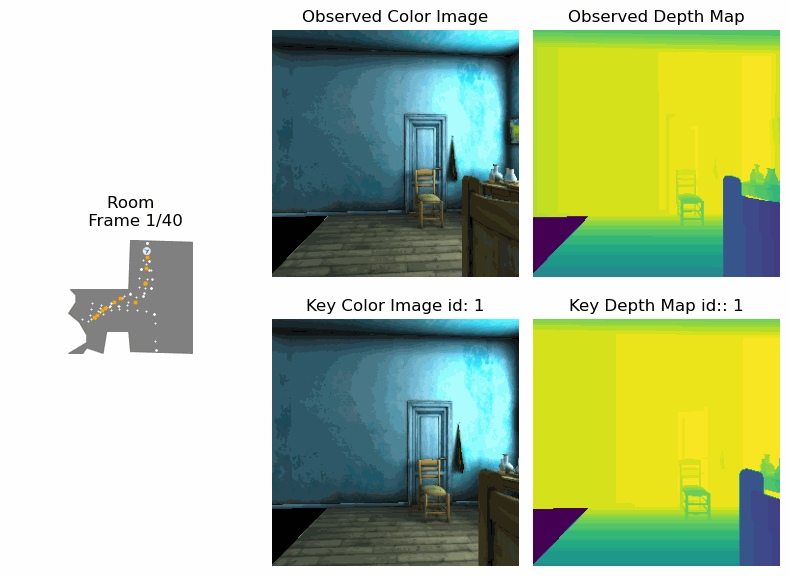

Supplement: Supplementary file 1 [file entropy-27-00641-s001.zip › supplementary_material/task-5.gif]

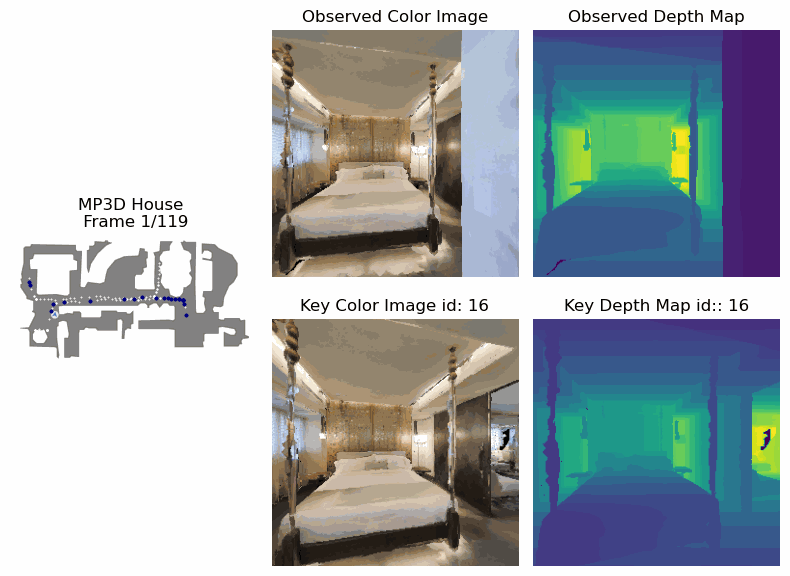

Supplement: Supplementary file 1 [file entropy-27-00641-s001.zip › supplementary_material/task-8.gif]

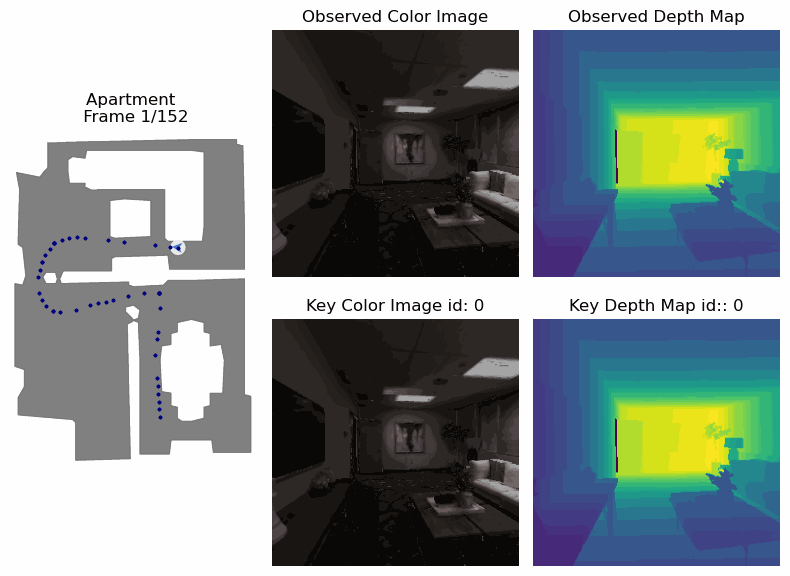

Supplement: Supplementary file 1 [file entropy-27-00641-s001.zip › supplementary_material/task-2.gif]

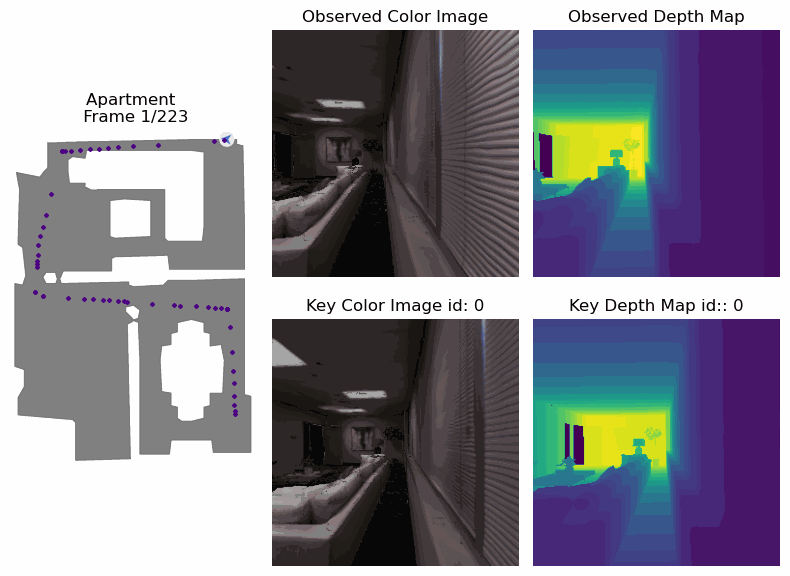

Supplement: Supplementary file 1 [file entropy-27-00641-s001.zip › supplementary_material/task-1.gif]

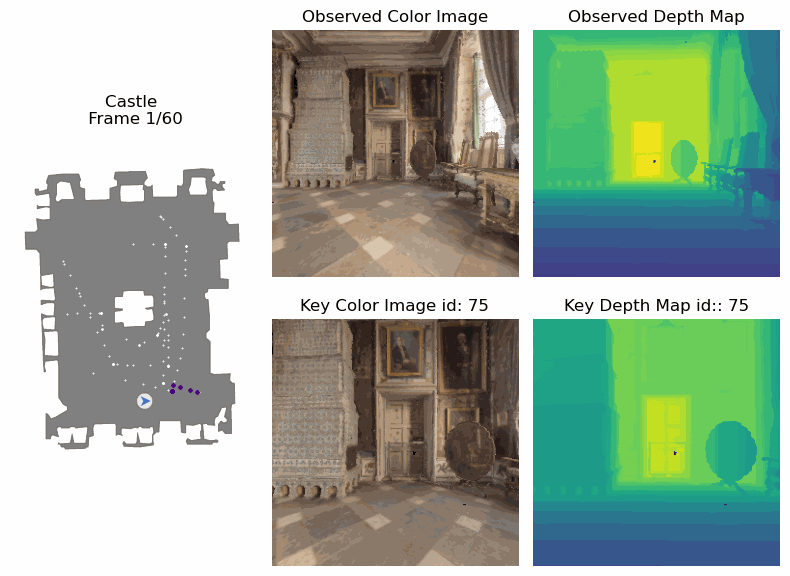

Supplement: Supplementary file 1 [file entropy-27-00641-s001.zip › supplementary_material/task-6.gif]

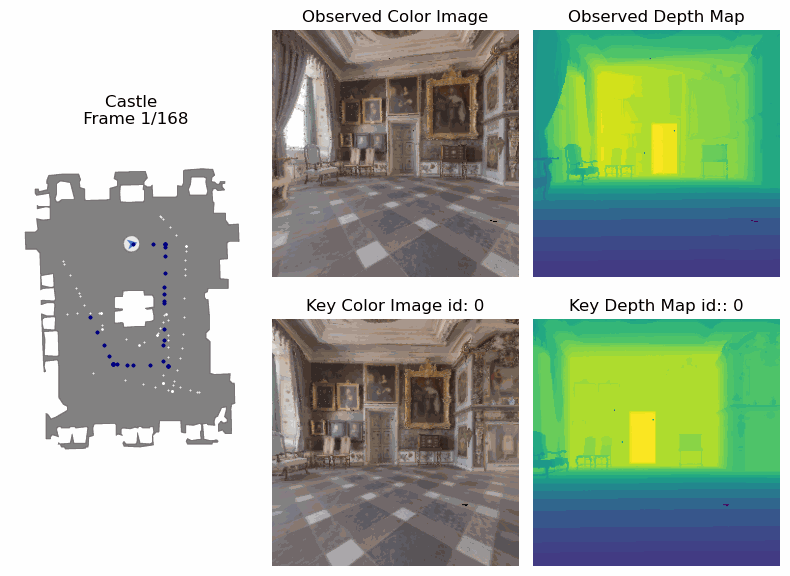

Supplement: Supplementary file 1 [file entropy-27-00641-s001.zip › supplementary_material/task-7.gif]
